# Physiological significance of *WDR45*, a responsible gene for β-propeller protein associated neurodegeneration (BPAN), in brain development

**DOI:** 10.1038/s41598-021-02123-3

**Published:** 2021-11-19

**Authors:** Mariko Noda, Hidenori Ito, Koh-ichi Nagata

**Affiliations:** 1 Department of Molecular Neurobiology, Institute for Developmental Research, Aichi Developmental Disability Center, 713-8 Kamiya, Kasugai, 480-0392 Japan; 2 Department of Neurochemistry, Nagoya University Graduate School of Medicine, Nagoya, Japan

**Keywords:** Disease model, Diseases of the nervous system, Neuroscience

## Abstract

WDR45 plays an essential role in the early stage of autophagy. De novo heterozygous mutations in *WDR45* have been known to cause β-propeller protein-associated neurodegeneration (BPAN), a subtype of neurodegeneration with brain iron accumulation (NBIA). Although BPAN patients display global developmental delay with intellectual disability, the neurodevelopmental pathophysiology of BPAN remains largely unknown. In the present study, we analyzed the physiological role of Wdr45 and pathophysiological significance of the gene abnormality during mouse brain development. Morphological and biochemical analyses revealed that Wdr45 is expressed in a developmental stage-dependent manner in mouse brain. Wdr45 was also found to be located in excitatory synapses by biochemical fractionation. Since *WDR45* mutations are thought to cause protein degradation, we conducted acute knockdown experiments by in utero electroporation in mice to recapitulate the pathophysiological conditions of BPAN. Knockdown of *Wdr45* caused abnormal dendritic development and synaptogenesis during corticogenesis, both of which were significantly rescued by co-expression with RNAi-resistant version of Wdr45. In addition, terminal arbors of callosal axons were less developed in Wdr45-deficient cortical neurons of adult mouse when compared to control cells. These results strongly suggest a pathophysiological significance of *WDR45* gene abnormalities in neurodevelopmental aspects of BPAN.

## Introduction

Autophagy is a highly dynamic and complex process essential for cell growth, survival, development and death^1,2^. Autophagy involves a sequential set of events including autophagosome formation, maturation, fusion with lysosomes, subsequent breakdown and the release of macromolecules back into the cytosol^2^. WDR45 (also known as WIPI4), an evolutionary conserved β-propeller scaffold protein, is a component of the autophagy machinery and controls an early step of autophagosome formation^3^.

De novo heterozygous mutations in *WDR45* have recently been shown to cause β-propeller protein-associated neurodegeneration (BPAN), also known as static encephalopathy of childhood with neurodegeneration in adulthood (SENDA)^4^, which is a subtype of neurodegeneration with brain iron accumulation (NBIA)^5,6^. Typical presentation of BPAN consists of global developmental delay in early childhood with intellectual disability (ID), with or without concurrent spasticity, with patients subsequently developing sudden onset of progressive dystonia, parkinsonism and dementia during their 20 s to early 30 s. In the course of clinical deterioration, iron accumulation becomes evident in the substantia nigra and globus pallidus based on brain imaging^7^. Since WDR45 has a crucial role in autophagy, an impaired autophagy mechanism has been considered to underlie the etiology of BPAN. Indeed, the autophagic flux, the whole process of autophagy, has been reported to be reduced in lymphoblastoid cell lines (LCLs) from BPAN patients^4^. Also, WDR45 protein expression was severely reduced in LCLs derived from all BPAN patients analyzed, strongly suggesting that *WDR45* mutations make the protein unstable and undergo degradation^4^, and finally disturb autophagosome formation and subsequent autophagic processes. Mutations in *WDR45* also have genetic overlaps with those from neurodevelopmental disorder patients with ID and epileptic encephalopathy^8^, indicative of an essential role of WDR45 during brain development.

In this study, we tried to clarify the pathophysiological significance of *WDR45* gene abnormalities in neurodevelopmental delay, especially ID, observed in infancy of BPAN patients. To this end, we first performed expression analyses and found that Wdr45 is expressed in mouse brain in a developmental stage-dependent manner. Subsequent functional analyses revealed essential roles of Wdr45 in dendrite development, axon pathfinding and synapse formation. These results indicate the physiological relevance of WDR45 during brain development and potential involvement of its functional deficiency in the clinical features of neurodevelopmental aspects of BPAN.

## Results

### Expression profile of Wdr45 protein in mouse tissues

**Figure 1:**
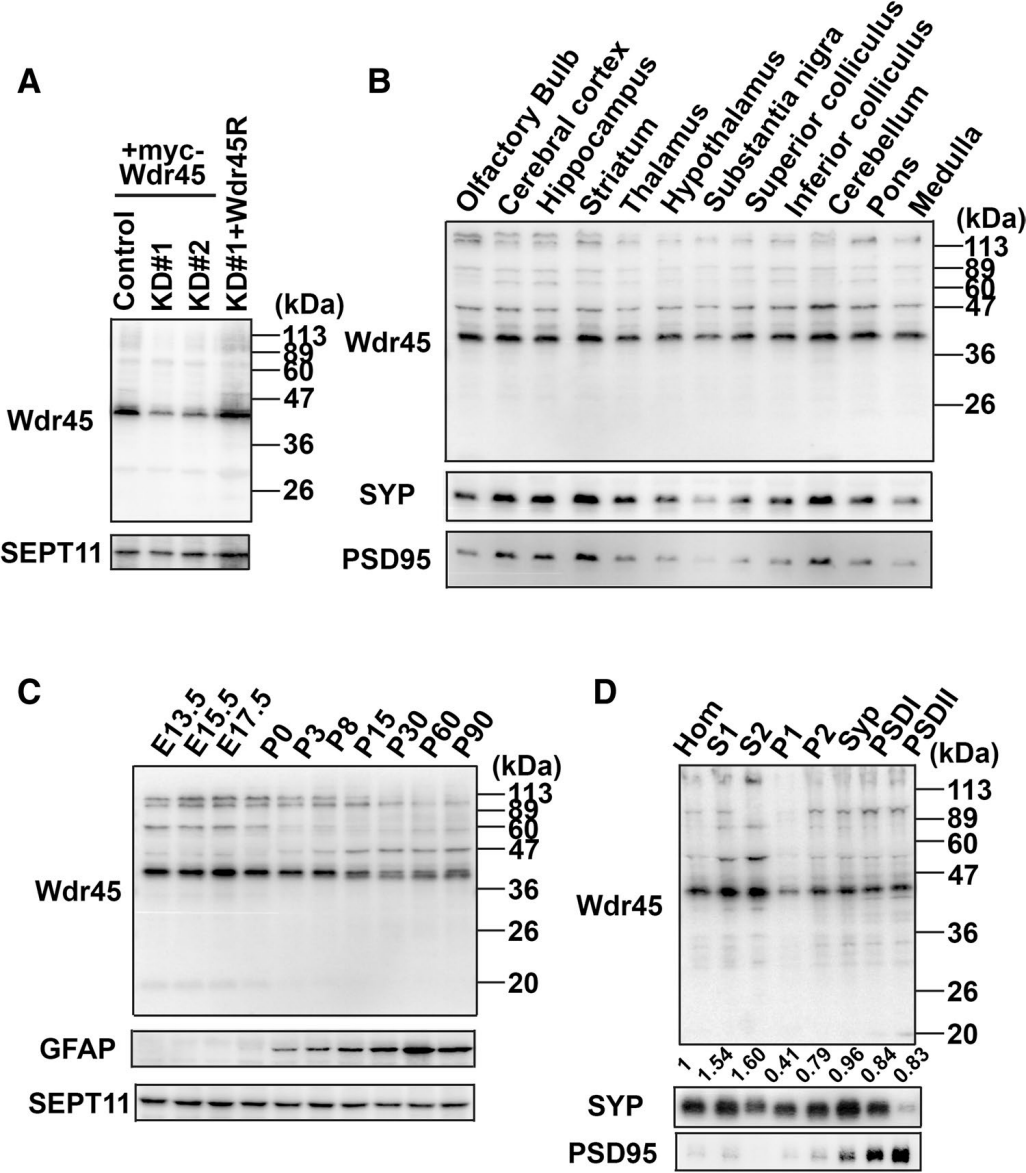
Expression profiles of Wdr45 in mouse tissues. (**A**) Characterization of anti-Wdr45. COS7 cells were transfected with pSuper-Luc (control), pSuper-Wdr45#1(KD#1) or -Wdr45#2 (KD#2) together with pCAG-Myc-Wdr45 or -Myc-Wdr45R. After 48 h, cells were harvested and lysates were subjected to western blotting using anti-Wdr45. The blot was probed with anti-Sept11 as loading control. (**B**) Regional expression of Wdr45 in brain. Various regions were dissected from adult mouse and whole tissue extracts were prepared as in “Materials and methods” section. Each extract (20 µg of proteins) was subjected to western blotting using anti-Wdr45, anti-synaptophysin and anti-PSD95. (**C**) Whole brain lysates (30 µg protein *per* lane) at indicated developmental stages were subjected to western blotting with anti-Wdr45. GFAP and Sept11 were used as differentiation marker and loading control, respectively. **(D**) Subcellular fractions (30 µg protein *per* lane) prepared from adult forebrains by differential and density-gradient centrifugation were subjected to western blotting with anti-Wdr45. Anti-synaptophysin (SYP) and anti-PSD95 were used as presynaptic and postsynaptic markers, respectively. The relative levels of the ~ 40 kDa band were measured by densitometry and shown as fold-increase compared to the expression level of the homogenate. *Hom* homogenate, *S1* soluble fraction, *S2* crude cytosol fraction, *P1* nuclear fraction, *P2* crude membrane fraction, *Syp* synaptosomal fraction, *PSDI* PSD fraction-I, *PSDII* PSD fraction-II. Uncropped blots are shown in Supplementary Figs. S6–S9.

As shown in Fig. 1A, anti-Wdr45 detected Myc-tagged Wdr45 (Myc-Wdr45) with ~ 40 kDa expressed in COS7 cells in western blotting. The immunoreactivity was significantly reduced when Myc-Wdr45 was knocked down with pSuper-Wdr45#1 or -Wdr45#2 (Fig. 1A). To characterize the expression of Wdr45 in brain, whole tissue extracts were prepared from various brain regions of adult mouse and subjected to western blot analyses using anti-Wdr45 (Fig. 1B). As a control experiment, the distribution pattern of synaptophysin was analyzed and found to be very similar to what previously described^9^. Wdr45 with a molecular mass of ~ 40 kDa was detected in all the regions including basal ganglia where iron deposition is generally observed in BPAN patients, although striatum and substantia nigra in basal ganglia contained relatively low levels of the protein (Fig. 1B). To gain some insight into the involvement of Wdr45 in neuronal development, we next analyzed its expression level in whole brain extracts at various developmental stages. Wdr45 (at ~ 40 kDa) was strongly detected during embryonic stages and then gradually decreased after postnatal day (P)8 to the lowest level at P90 (Fig. 1C). Approximately 110 kDa and ~ 60 kDa bands also showed weak expression patterns similar to that of the ~ 40 kDa protein, whereas weak expression of a ~ 50 kDa band appeared after birth.

When the Wdr45 expression profile was analyzed in peripheral mouse tissues, the ~ 40 kDa band was detected in all tissues except small intestine (Supplementary Fig. S1). While this ~ 40 kDa band is abundant in brain, liver, spleen, pancreas, ovary and uterus, it was weakly detected in heart, adrenal gland and skeletal muscle. A ~ 50 kDa band was detected in brain, heart and skeletal muscle, and an ~ 85 kDa protein was relatively well expressed in liver and kidney. Meanwhile, a ~ 110 kDa band was strongly and moderately expressed in pancreas and liver, respectively. In addition, tissue-specific bands with ~ 60 kDa, ~ 70 kDa and ~ 28 kDa were observed in brain, pancreas and skeletal muscle, respectively (Supplementary Fig. S1). It is, however, possible that some of these bands are non-specific or represent degradation products during sample preparation. Further genetic analyses of mouse *Wdr45* gene as well as analyses with isoform-specific antibodies should be required.

Although not highly expressed in adult brain, WDR45 might be distributed in synapses and functional defects there might be related to the clinical symptoms of BPAN. We therefore performed biochemical fractionation of adult mouse brain tissue, and the quality of each fraction was confirmed by detection of synaptophysin and PSD95, markers for pre- and post-synapses, respectively (Fig. 1D). Consequently, the ~ 40 kDa and ~ 80 kDa proteins, but not the ~ 50 kDa one, were detected in highly purified post synaptic density (PSD) (PSD-II) fraction where synaptophysin was very weakly detected (Fig. 1D). The ~ 110 kDa and ~ 50 kDa proteins were only faintly observed in the PSD fraction (Fig. 1D). These results suggest that certain Wdr45 isoforms are distributed at PSD. Based on densitometry analysis, ~ 0.91% of the total amount of the ~ 40 kDa protein was estimated to be present in the purified PSD-I and -II fractions. Given that the synaptosome fraction contains both pre- and post-synaptic components and PSD-I fraction contains contaminated presynaptic proteins, Wdr45 also might be present in the pre-synaptic compartment. Taken together, these results suggest that Wdr45 could be involved in synapse structure and/or function in differentiated neurons given its distribution.

### Immunohistochemical analysis of Wdr45 in mouse brain

**Figure 2:**
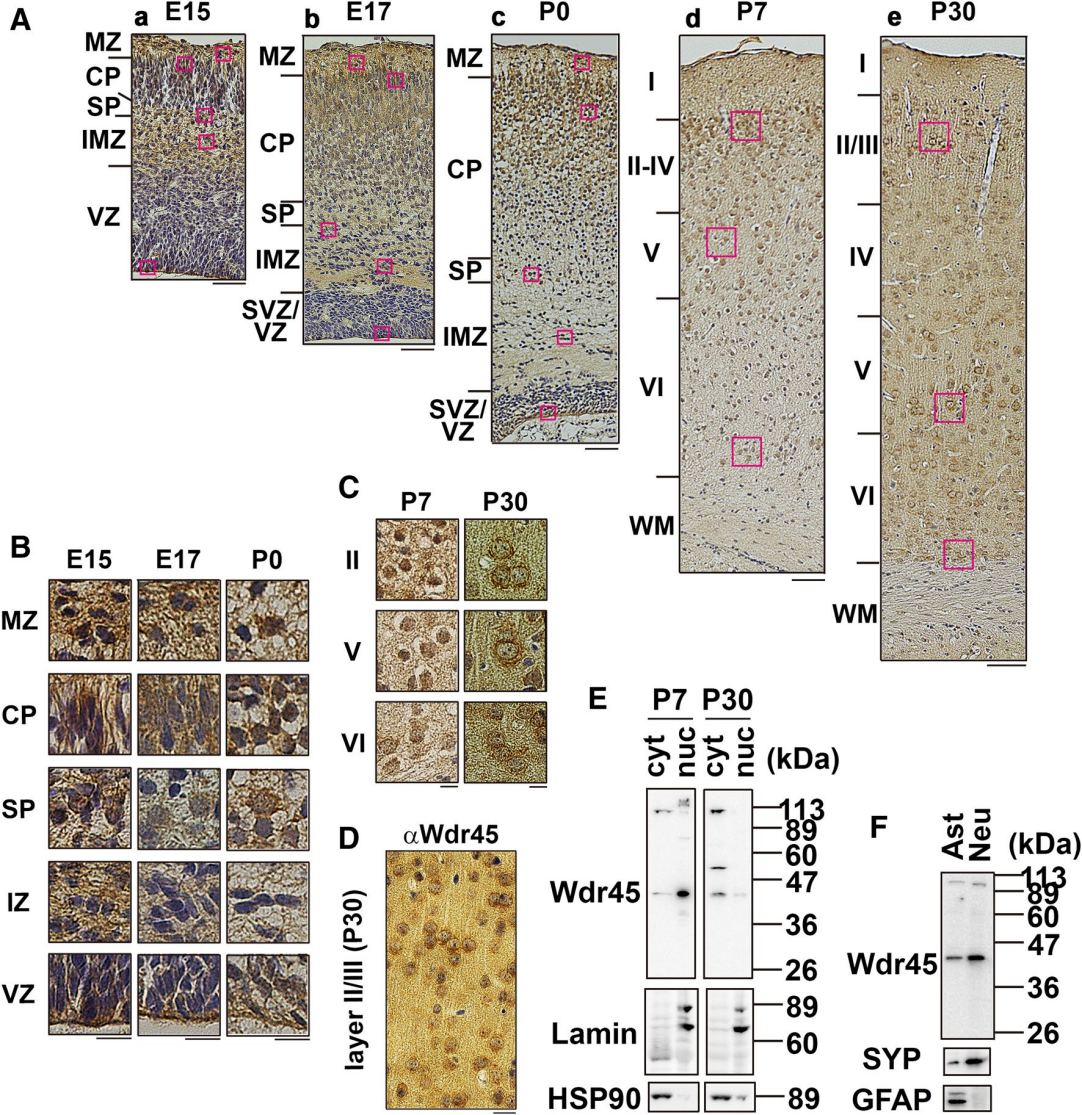
Immunohistochemical analysis of Wdr45 in mouse cerebral cortex. (**A**) Coronal sections of the somatosensory area at E15 (a), E17 (b), P0 (c), P7 (d) and P30 (e) were stained with anti-Wdr45. *VZ* ventricular zone, *SVZ* subventricular zone, *IMZ* intermediate zone, *SP* subplate, *CP* cortical plate, *MZ* marginal zone, *WM* white matter. (**B**) Cells at the indicated areas in ((**A**), a–c) were magnified. (**C**) Cells at the indicated areas in ((**A**), d,e) were magnified. (**D**) An area of Layer II/III in the cortex at P30 was co-stained with anti-Wdr45. Counter staining was performed using Mayer’s hematoxylin. Scale bars, 50 µm (**A**) and 10 µm (**B–D**). (**E**) The cytoplasmic (20 μg of protein) and nuclear (20 μg; proportionally assigned amount corresponding to the cytoplasmic proteins used) fractions from P7 and P30 cerebra were immunoblotted with anti-Wdr45. The fractions were also immunoblotted with anti-lamin and anti-HSP95 to confirm proper fractionation. (**F**) Cell lysates (20 mg) of cultured astrocytes (Ast) and cortical neurons (Neu) were immunoblotted with anti-Wdr45. The membrane was then re-probed with anti-synaptophysin (SYP) and anti-GFAP. Uncropped blots are shown in Supplementary Figs. S10 and S11.

To determine the localization of Wdr45, we performed immunohistochemical analyses of cerebral cortex at embryonic day (E) 15, E17, P0, P7 and P30. At E15 and E17, Wdr45 was broadly detected in neurons in the marginal zone (MZ), cortical plate (CP), subplate (SP) and intermediate zone (IMZ) as well as neuronal progenitors in the ventricular zone (VZ) and subventricular zone (SVZ) (Fig. 2A,a,b,B). Although the broad distribution pattern was observed until P30, subcellular distribution changed after P7; relative nuclear enrichment of Wdr45 was frequently observed from E15 to P7 while cytoplasmic distribution clearly appeared at P30 (Fig. 2A,e,C). Notably, Wdr45 was also detected at the ventricular surface of neural progenitors from E15 to P0 (Fig. 2B). In addition, distinct immunoreactivity was observed in the neuropil of cerebral cortex at P30 (Fig. 2D), strongly suggesting enrichment at excitatory synapses. Since Wdr45 changed its distribution during cortical development, we prepared the nuclear and cytoplasmic fractions from cerebra at P7 and P30. While Wdr45 was predominantly detected in the nuclear fraction at P7, it was mainly detected in the cytoplasm at P30 (Fig. 2E). Collectively, Wdr45 was expressed in the nucleus of cortical neurons and the ventricular surface during corticogenesis and its subcellular localization changed from the nucleus to the cytoplasm and then neuropils in developed cerebral cortex. We also confirmed that Wdr45 was predominantly expressed in cortical neurons when compared to astrocytes (Fig. 2F).

**Figure 3:**
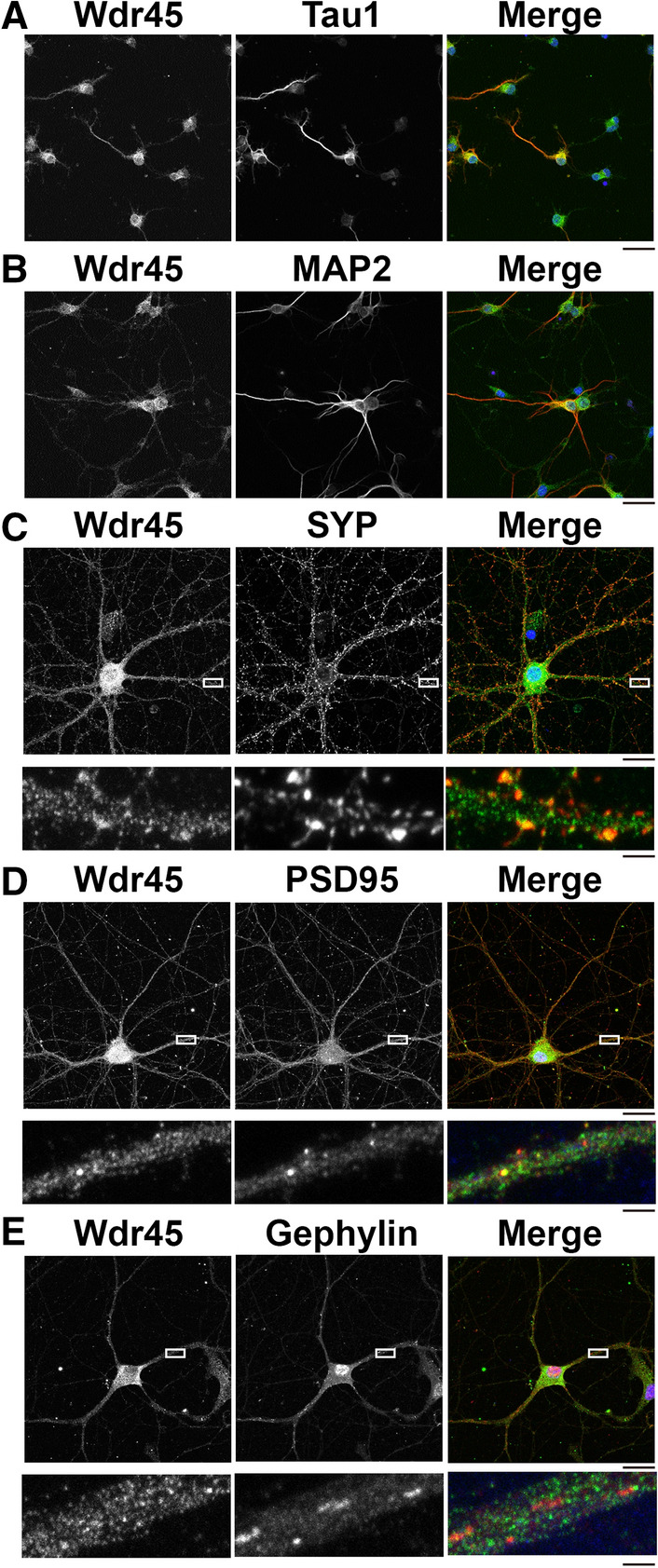
Localization of Wdr45 in primary cultured mouse hippocampal neurons. (**A,B**) Neurons cultured for 3 (**A**) or 7 days (**B**) in vitro were double-stained for Wdr45 with Tau-1 (**A**) or MAP2 (**B**). Merged images were also shown. Scale bars, 20 µm. (**C**–**E**) Neurons cultured for 14 days were double-stained for Wdr45 with synaptophysin (**C**), PSD95 (**D**) or gephyrin (**E**). Boxed areas in the *upper* panels were magnified in the *lower* panels. Scale bars, 20 µm (*upper* panels) and 5 µm (*lower* panels).

Since Wdr45 is expressed in adult mouse central nervous system (CNS), it might play a role in differentiated neurons. We thus examined the expression of Wdr45 in primary cultured hippocampal neurons during differentiation. Wdr45 was mainly observed in the nucleus and soma in immature neurons at 3 days in vitro (div) and 7 div (Fig. 3A,B). Wdr45 was also detected in Tau-1-positive axon and MAP2-positive dendrites (Fig. 3A,B). In 14 div neurons, in addition to the nucleus and cytoplasm, Wdr45 was visualized in dendrites, and partially colocalized with synaptophysin (a presynaptic marker) and PSD-95 (a postsynaptic excitatory neuron marker), but not gephyrin (an inhibitory synaptic marker) (Fig. 3C–E, *lower* panels). Together with the biochemical fractionation data (Fig. 1D), we assume that Wdr45 may participate in synaptic structure and/or functions in the hippocampal neurons.

Histological analyses of a BPAN patient with a loss-of-function mutation (p.Leu232Alafs*53) in *WDR45* showed mild atrophy and Tau-positive neurofibrillary tangles in hippocampus, and mild Purkinje cell depletion in cerebellum^6^. It is also notable that cerebellar atrophy was observed in ~ 25% of BPAN patients^7^. As for the animal model, CNS-specific Wdr45-knockout (KO) mice have been shown to recapitulate some hallmarks of BPAN such as cognitive impairment, strongly suggesting that Wdr45-deficiency influences the function of hippocampus^10^. In addition, poor motor coordination was observed in the KO mice, suggesting impairment of cerebellar functions controlling coordination, precision and accurate timing of movements^10^. We thus carried out immunohistochemical analyses in hippocampus at E17, P0, P7 and P30 and in cerebellum at P30 (Supplementary Fig. S2). As in the case of cerebral cortex, Wdr45 appeared to be expressed in the nucleus and cytoplasm at E17 and P0 in the CA regions and dentate gyrus of hippocampus (Supplementary Fig. S2A,B). Then, the cytoplasmic distribution in the neurons of CA regions as well as dentate gyrus came to be detected at P7 and became clearer at P30 (Supplementary Fig. S2C,D). In cerebellum, almost all regions were positive for Wdr45 (Supplementary Fig. S2E). It is of note that Wdr45 appeared to be enriched in the nucleus of Purkinje cells at P30, in contrast to the cytoplasmic distribution in cortical neurons (Supplementary Fig. S2E, Fig. 2A,e). Specificity of the anti-Wdr45 in immunohistochemistry was confirmed in Supplementary Fig. S3. These results are consistent with possible roles of Wdr45 in hippocampus and cerebellum.

### Characterization of RNAi- and expression-vectors

**Figure 4:**
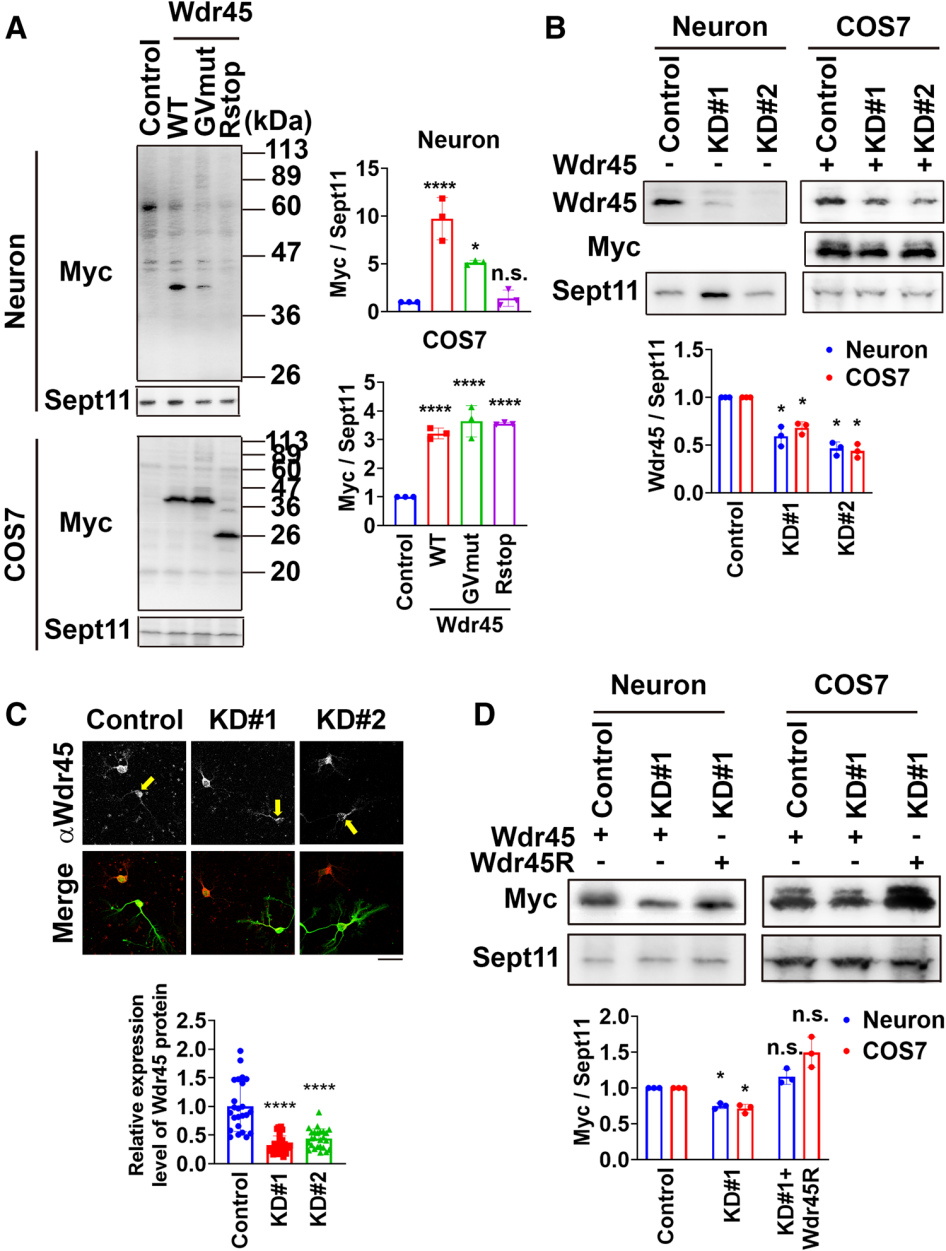
Characterization of vectors for expression and RNAi. (**A**) Expression of wild type Wdr45 and 2 mutants, c.700C > T/p.(Arg234*) (Wdr45-Rstop) and c.439G > T/p.(Gly167Val) (GVmut), in dissociated mouse cortical neurons or COS7 cells. Neurons isolated at E14 and COS7 cells were transfected with pCAG vector (control), pCAG-Myc-Wdr45 (WT), -Wdr45-GVmut or -Wdr45-Rstop, and cultured in vitro for 13 days (neurons) and cultured for 48 h (COS7 cells). Cells were then subjected to western blotting with anti-Myc. Anti-Sept11 was used for a loading control. For quantification, Myc-band intensities were quantified densitometrically and normalized against the loading control. The values were plotted and error bars indicate SD (n = 3); *p < 0.05, ****p < 0.0001, *n.s.* means not significant by one-way ANOVA with Tukey’s post hoc test. (**B**) Knockdown of endogenous Wdr45 in dissociated mouse cortical neurons or exogenous Wdr45 in COS7 cells. Neurons were co-transfected with pCAG-EGFP together with pSuper-H1.shLuc (Control), pSuper-Wdr45#1 (KD#1) or #2 (KD#2). COS7 cells were co-transfected with pCAG-Myc-Wdr45 together with pSuper-H1.shLuc (Control), pSuper-Wdr45#1 (KD#1) or #2 (KD#2). After culture for 13 days and 48 h for neurons and COS7 cells, respectively, cells were harvested and subjected to western blotting with anti-Wdr45 or anti-Myc. Anti-SEPT11 was used for loading control. Band intensities were quantified as in (**A**). The values were plotted and error bars indicate SD (n = 3); *p < 0.05 by one-way ANOVA with Tukey’s post hoc test. (**C**) Immunostaining of dissociated cortical neurons. pCAG-EGFP was co-transfected with pSuper-H1.shLuc (Control), pSuper-Wdr45#1 or #2 into neurons as in (**A**), and cultured for 72 h. After fixation, cells were immunostained with anti-GFP (green) and anti-Wdr45 (red). Wdr45 expression levels were estimated by measurement of fluorescence intensity. The values normalized to the control average value were plotted. Error bars indicate SD; n = 3 experiments involving independent batches of neurons cultured from different embryos. At least 10 neurons were analyzed for each group; ****p < 0.0001 by one-way ANOVA with Dunnett’s post hoc test. Scale bars, 50 μm. (**D**) Characterization of RNAi-resistant Wdr45. pCAG-Myc-Wdr45 or pCAG-Myc-Wdr45R was co-transfected into dissociated mouse cortical neurons or COS7 cells with pSuper-H1.shLuc (Control), pSuper-Wdr45#1 or #2. Cells were further cultured and then subjected to western blotting as in (**B**) Myc-band intensities were quantified as in (**B**). The values were plotted and error bars indicate SD (n = 3); *p < 0.05, *n.s.* means not significant by one-way ANOVA with Tukey’s post hoc test. Uncropped blots are shown in Supplementary Figs. S12–S14.

Given that *WDR45* gene abnormalities are responsible for the early onset of ID in BPAN, Wdr45 is most likely to participate in the formation of cortical architecture. BPAN-causative mutants of WDR45 are supposed to be structurally unstable and undergo degradation^4^. Indeed, a BPAN-causative mutant, c.700C > T/p.(Arg234*) (Wdr45-Rstop), as well as another mutant, c.439G > T (GVmut), with single amino acid substitution in the linker region between third and fourth β-propeller domains were hardly detected when expressed in primary cultured mouse cortical neurons, strongly suggesting that haploinsufficiency of *WDR45* underlies the pathogenesis of BPAN (Fig. 4A). It is of note that Wdr45-Rstop and GVmut did not undergo degradation in COS7 cells, which might explain the CNS-dominant clinical feature of BPAN. We thus conducted acute knockdown experiments to recapitulate the pathophysiological conditions in BPAN. To this end, 2 RNAi vectors were designed against mouse Wdr45, pSuper-Wdr45#1 and #2, both of which effectively knocked down not only Myc-Wdr45 expressed in COS7 cells but also endogenous Wdr45 in primary cultured cortical neurons (Fig. 4B). Immunostaining analyses confirmed the knockdown of endogenous Wdr45 in cortical neurons in vitro (Fig. 4C). An RNAi-resistant version of Wdr45, Wdr45R, was shown to be resistant against pSuper-Wdr45#1 (Fig. 4D).

### Role of Wdr45 in cortical neuron migration

We asked whether Wdr45 is involved in the migration of newly generated cortical neurons by in utero electroporation-based acute knockdown of Wdr45. pSuper-H1.shLuc (control), pSuper-Wdr45#1 or #2 was co-electroporated with pCAG-Turbo RFP into VZ progenitor cells, and electroporated brains were fixed and analyzed at P2. When compared to the control experiments, Wdr45-knockdown had little effects on the migration and the cells were considered to normally make it to the layer II-IV (Supplementary Fig. S4).

### Role of Wdr45 in axon extension and dendritic arbor development during brain development

**Figure 5:**
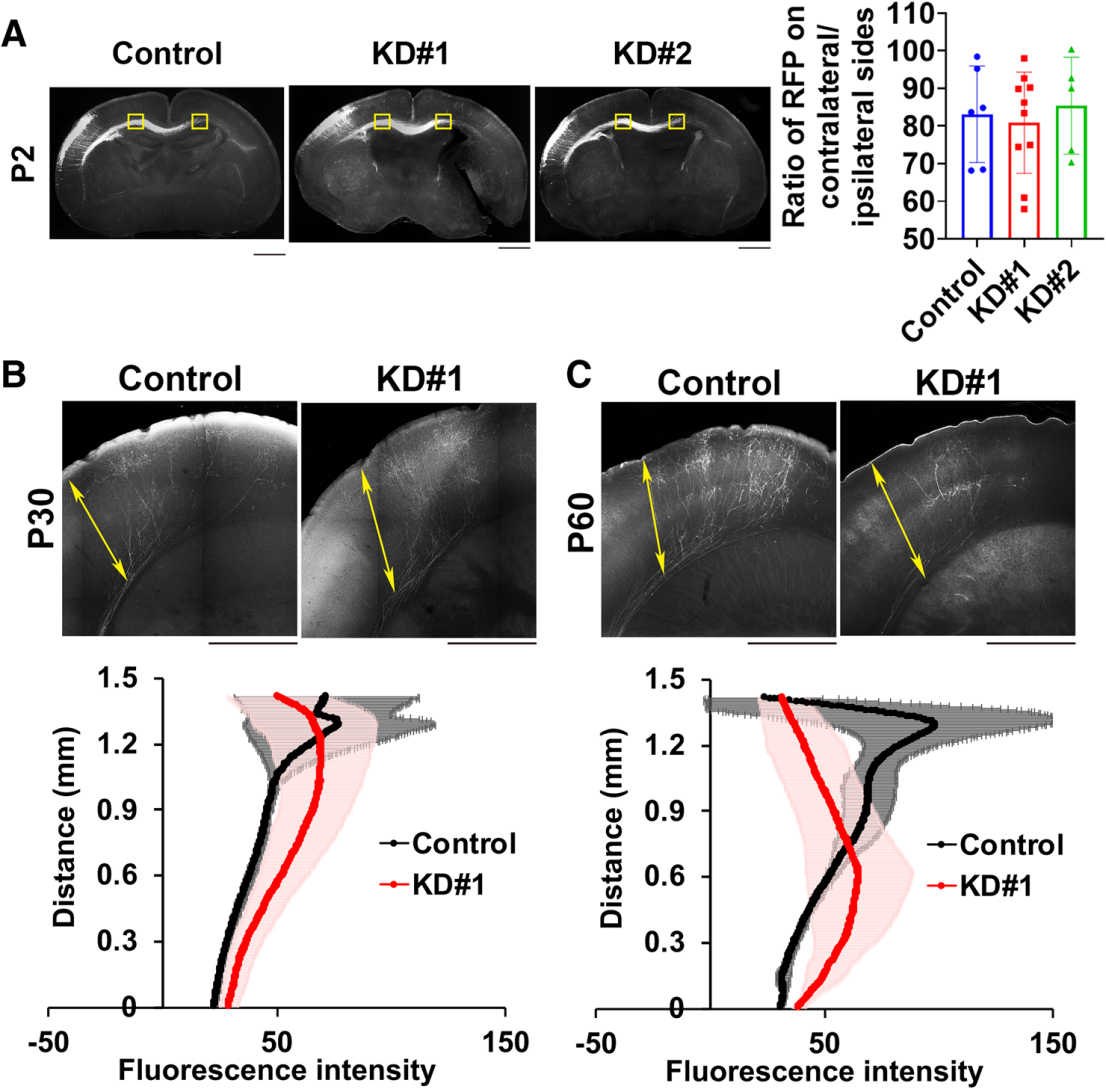
Role of Wdr45 in the callosal axon growth in vivo. (**A**) pCAG-Turbo-RFP was co-electroporated with pSuper-H1.shLuc (control), pSuper-Wdr45#1 or #2 into the VZ progenitor cells at E14.5 and fixed at P2. Coronal sections were then prepared and analyzed. Representative images of callosal axons expressing RFP were shown. Note that Wdr45-deficiency less affected the axon growth at this timing. Quantitative analyses of the ratio of the intensity of RFP-positive axons in the area (yellow) of contralateral cortex to that of ipsilateral one. Error bars indicate SD; Control (n = 6), KD#1 (n = 10), KD#2 (n = 5) from 3 independent experiments; not significant by one-way ANOVA with Tukey’s post hoc test. (**B,C**) Effects of Wdr45-knockdown on axon extension into the contralateral cortex at P30 (**B**) or P60 (**C**). Representative images of the terminal arbors of callosal axons expressing RFP with pSuper-H1.shLuc or pSuper-Wdr45#1 were shown. Quantitative densitometric analyses of RFP fluorescence intensity were performed for axons extending into the contralateral cortical layer structure of control (black line) and Wdr45-deficient (red line) neurons. Distance from the VZ surface along *double sided arrows* represents the vertical (y) axis. Shadows represent SD [control, n = 3 and KD#1, n = 6 for (**B**); control, n = 3 and KD#1, n = 5 for (**C**)]. Scale bars, 50 mm (**A**) and 500 µm (**B**).

Since aberrant synaptic network formation is tightly associated with defective brain development and function, Wdr45 may take part in axon pathfinding and/or dendrite growth during brain development. We thus first examined the interhemispheric axon projection of Wdr45-deficient cortical neurons in vivo. To easily detect morphological changes, we analyzed pyramidal neurons in layer II/III which are generated at E14–16. To this end, Wdr45 was knocked down in the VZ cells at E14.5. Consequently, axon bundles from the hemisphere containing Wdr45-deficient pyramidal neurons normally reached the contralateral hemisphere at P2 (Fig. 5A) and this tendency was still observed at P30 (Fig. 5B). On the other hand, it is notable that axons of the Wdr45-deficient cells did not penetrate efficiently into the layer structure of contralateral cortex at P60 when compared to control axons (Fig. 5C). Overall, Wdr45 appears to be important for callosal axon extension into the cortical structure after normal pathfinding from the ipsilateral to contralateral cortex.

**Figure 6:**
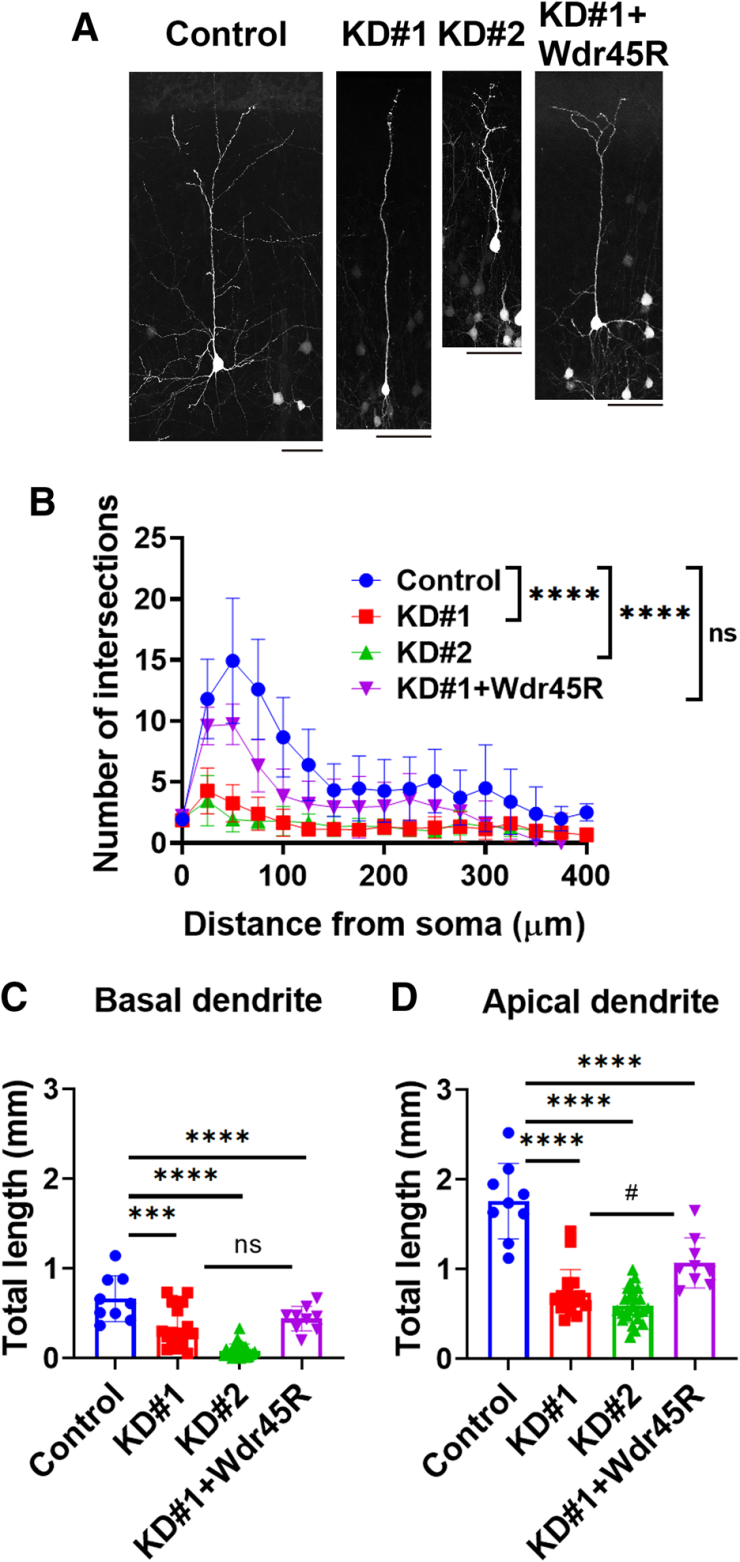
Effects of Wdr45-knockdown on dendrite growth in vivo. (**A**) For sparse expression, pCAG-loxP-GFP (0.5 µg) was co-electroporated with pCAG-M-Cre (1 ng) together with pSuper-H1.shLuc (control), pSuper-Wdr45#1, #2 or pSuper-Wdr45#1 plus pCAG-Myc-Wdr45R (1.5 µg each of pSuper vector and 0.5 µg of pCAG-Myc-Wdr45R) at E14.5. Coronal sections were prepared, stained for GFP and analyzed at P10. Representative average z-stack projection images of layer II/III cortical neurons were shown. Scale bars, 50 μm. (**B**) Branch points of dendrites in (**A**) were quantified by Sholl test. One section from each brain was analyzed for control (n = 3), KD#1 (n = 7), KD#2 (n = 6), and KD#1 + Wdr45R (n = 5). Error bars indicate SD of the results from 10 to 15 neurons; ****p < 0.0001, by one-way ANOVA with Dunnett’s post hoc test (vs. control). (**C,D**) Total length was calculated for basal (**C**) and apical dendrites (**D**). ^#^*p* < 0.05, ***p < 0.001 and ****p < 0.0001 (*vs control; ^#^vs KD#1) by one-way ANOVA with Tukey’s post hoc test.

We then examined the knockdown effects on dendritic arbor development of cortical neurons in vivo. Electroporation of pSuper-Wdr45#1 or #2 into the VZ cells at E14.5 gave rise to highly suppressed dendritic arborization at P10 (Fig. 6A). Sholl analyses revealed a significant decrease in branch point number of both apical and basal dendrites in the deficient neurons at P10 (Fig. 6B). Rescue experiments were then performed to exclude off-target effects. When an RNAi-resistant version of Wdr45, Wdr45R, was co-electroporated into the VZ progenitor cells with pSuper-Wdr45#1, morphological defects were rescued, supporting the specificity of the effects caused by Wdr45 knockdown (Fig. 6A,B). We then measured the knockdown effects on the basal and apical dendrites, separately. As shown in Fig. 6C, Wdr45-knockdown by pSuper-Wdr45#1 or #2 suppressed basal dendrite development. In this analysis, while the phenotype tended to be rescued by Wdr45R, the effects were not statistically significant (Fig. 6C). Meanwhile, apically oriented dendritic length was abnormally shortened in pSuper-Wdr45#1- or #2-transfected cells at P10, and the phenotype by pSuper-Wdr45#1 was rescued at least partially (Fig. 6D). Collectively, we conclude that Wdr45-deficiency impairs neuronal connectivity through defective axon elongation as well as aberrant dendrite arborization.

### Role of Wdr45 in dendritic spine morphology regulation in vivo

**Figure 7:**
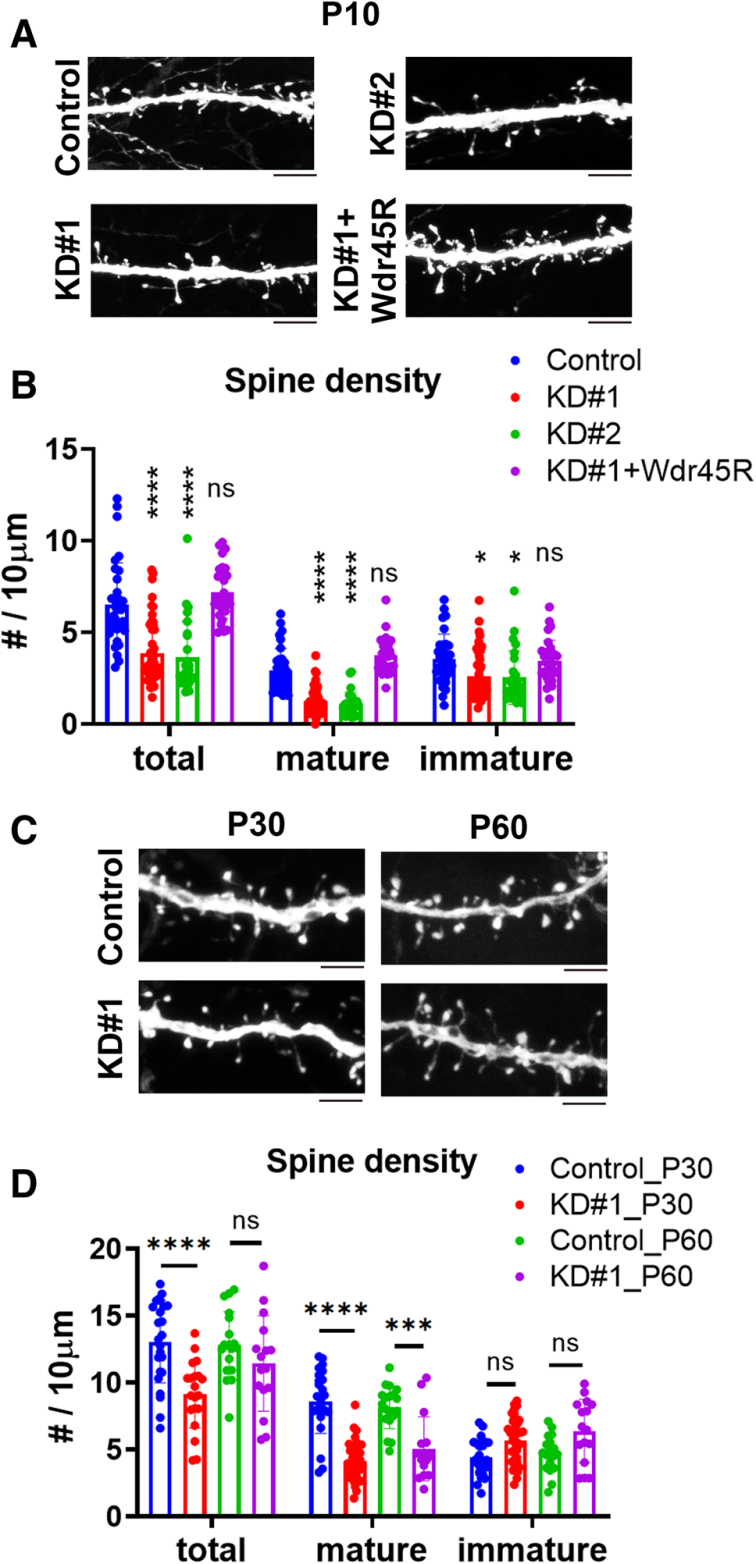
Effects of Wdr45-knockdown on the dendritic spine density and morphology in vivo. (**A**) pCAG-loxP-GFP was electroporated with pCAG-M-Cre together with pSuper-H1.shLuc (Control), pSuper-Wdr45#1 (KD#1), #2 (KD#2) or KD#1 plus pCAG-Myc-Wdr45R at E14.5. Coronal sections were prepared at P10 and stained for GFP. Representative images of dendritic spines on the 1st-order apical dendrites were shown. Scale bars, 5 μm. (**B**) Quantitative analyses of spine density and morphology (mature mushroom-shaped; immature filopodia-like or stubby spines) in layer II/III cortical excitatory neurons. One section from each brain was analyzed for control (n = 3), KD#1 (n = 4), KD#2 (n = 4) and KD#1 + Wdr45R (n = 5). Error bars indicate SD of the results from 20 to 30 neurons; *p < 0.05, ****p < 0.0001 by one-way ANOVA with Dunnett’s post hoc test (vs. control). (**C**) pCAG-loxP-GFP was co-electroporated with pCAG-M-Cre together with pSuper-H1.shLuc (Control) or pSuper-Wdr45#1 (KD#1) at E14.5. After fixation at P30 or P60, coronal sections were stained for GFP. Representative images of dendritic spines on the 1st-order apical dendrites were shown. Scale bars, 5 μm. (**D**) Quantitative analyses of spine density and morphology were performed as in (**B**). One section from each brain was analyzed for P30 (control, n = 5; KD#1, n = 6) and P60 (control, n = 4; KD#1, n = 5). Error bars indicate SD of the results from 20 to 30 neurons; ****p < 0.0001, ***p < 0.001 by one-way ANOVA with Tukey’s post hoc test (*vs control).

Dendritic spines are the primary site of excitatory input on most neurons, and changes in their shape and size are correlated with the strength of excitatory synaptic connections. Spine morphology can be classified into 3 groups, although there is a continuum of shapes between these categories: mushroom-type spine is mature while stubby-type and thin filopodia-like spines are immature. Mature spines form strong synaptic connections and have the longest lifetime, while filopodia-like spines show very mobile and flexible structures with a short lifetime^11^. Meanwhile, stubby spines typically do not have a neck and are likely to be formed during the disappearance of mature spines^12^. We here examined the physiological role of Wdr45 in the spine density and morphogenesis of layer II-III cortical neurons in vivo*.* To this end, pSuper-H1.shLuc (control), pSuper-Wdr45#1 or #2 was co-electroporated at E14.5 with pCAG-loxP-GFP plus pCAG-M-Cre for sparse GFP-labeling. Brains were then fixed and analyzed at P10. Total spine density was significantly decreased when Wdr45 was knocked down (Fig. 7A,B). The abnormal phenotype by pSuper-Wdr45#1 was rescued by co-expression of Wdr45R (Fig. 7A,B). In addition, knockdown of Wdr45 caused reduction of mature mushroom-type spine number (Fig. 7B). The phenotype by pSuper-Wdr45#1 was rescued by Wdr45R (Fig. 7B). We confirmed that Wdr45 was knocked down until P90 (Supplementary Fig. S5). We then examined the long-term effects of Wdr45-knockdown. While the reduced total spine density was still observed at P30, it was comparable to control neurons at P60 (Fig. 7C,D). In contrast, the number of mature mushroom-type spine remained lower at P60 (Fig. 7C,D). These results strongly suggest that Wdr45 plays a pivotal role in the synapse formation and/or maintenance.

### Long term effects of Wdr45-knockdown on neuronal morphology in vivo

**Figure 8:**
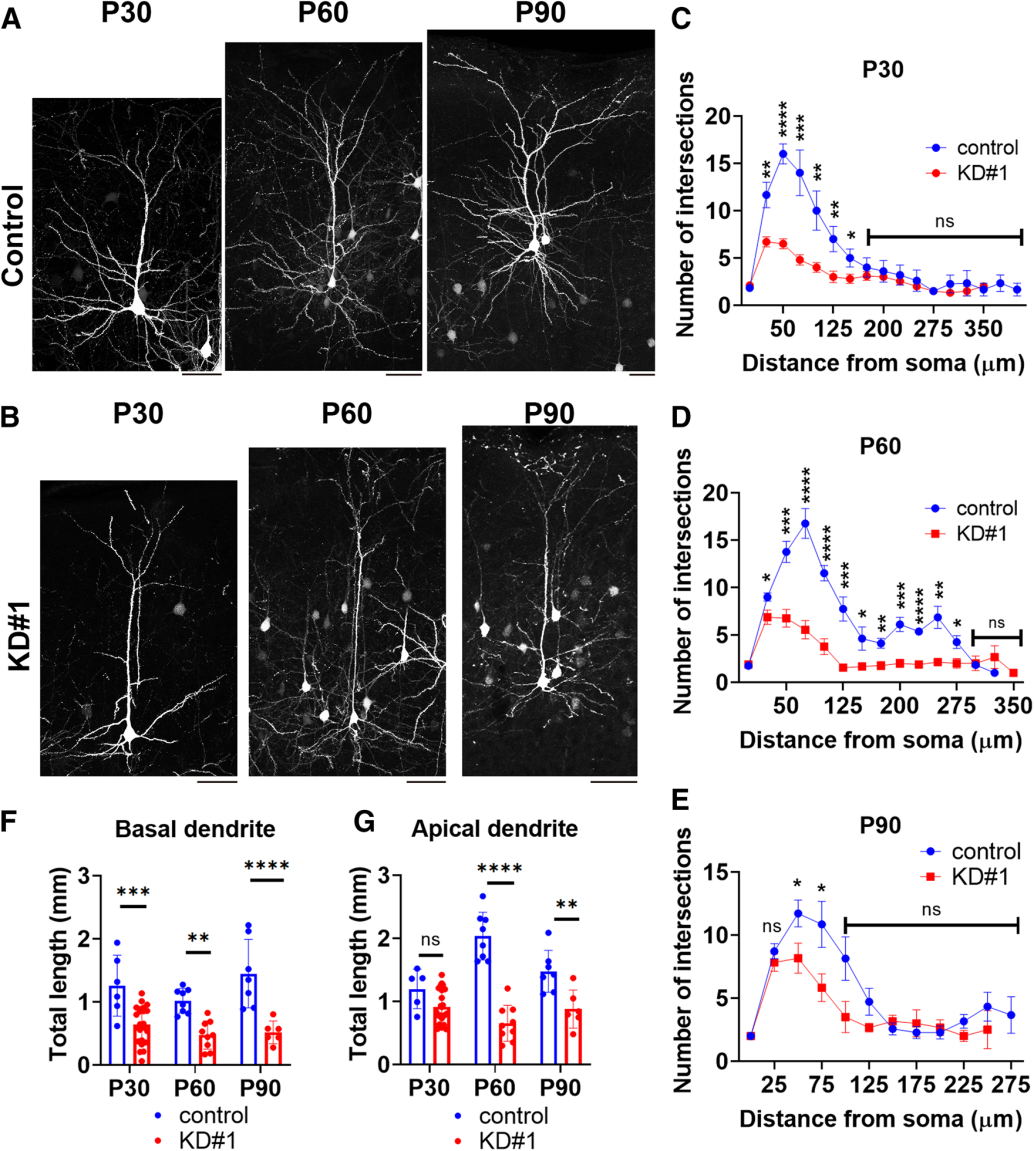
Long-term effects of Wdr45-knockdown on dendrite growth in vivo. (**A,B**) For sparse expression, pCAG-loxP-GFP was co-electroporated with pCAG-M-Cre together with pSuper-H1.shLuc (control) (**A**) or pSuper-Wdr45#1 (KD#1) (**B**) at E14.5. After fixation at P30, P60 or P90, coronal sections were prepared and stained for GFP. Representative average z-stack projection images of layer II/III cortical neurons were shown. Scale bars, 50 μm. (**C**–**E**) Branch points of dendrites were analyzed by Sholl test. One section from each brain was analyzed for P30 (control, n = 10; KD#1, n = 11) (**C**), P60 (control, n = 6; KD#1, n = 8) (**D**) and P90 (control, n = 3; KD#1, n = 3). Error bars indicate SD of the results from 10 to 15 neurons; *p < 0.05, **p < 0.01, ***p < 0.001 and ****p < 0.0001 by t-test (vs. control). (**F,G**) Total dendritic length was calculated for basal (**F**) as well as apical dendrites (**G**). Error bars indicate SD of the results from 10 to 15 neurons; *p < 0.05, **p < 0.01, ***p < 0.001 and ****p < 0.0001 by t-test (vs. control).

Although individuals with BPAN develop sudden-onset progressive dystonia, parkinsonism and dementia in adolescence, pathophysiological mechanism(s) underlying these clinical manifestations is yet to be clarified. To gain some insight into this aspect, we examined the long-term effects of Wdr45-knockdown on neuronal morphology by focusing on dendritic arbor development at P30, P60 and P90. To this end, we measured the intersection number of dendritic arbor as well as the total length of apical and basal dendrites. As shown in Fig. 8A–E, dendritic arbor complexity of Wdr45-deficient neurons remained poor when compared to control cells at the 3 time points mentioned above. As for dendritic arborization, basal dendrites of deficient neurons were shorter than that of the control cells at all time points tested (Fig. 8F). Meanwhile, apical dendrite length was also decreased at P60 and P90, whereas it was statistically unchanged at P30, albeit showing a tendency to decrease (Fig. 8G). Since apical dendrite development was inhibited at P10 (Fig. 6D), the development might recover temporarily around P30 during brain development, although its pathophysiological meaning is unclear. We confirmed that Wdr45 was knocked down at P30 and P90, indicating the possibility that knockdown effects lasted until the adult stage (Supplementary Fig. S5). These results strongly suggest that Wdr45-deficiency prevents neuronal network formation even in the adult stage and causes sustained impairment of synaptic function.

## Discussion

Autophagy, a basic process regulating intracellular degradation, is a key regulatory system for cell growth, differentiation, survival and physiological homeostasis^1,2,13,14^, and is thought to be impaired in various neurodegenerative disorders^13,15^. Given that autophagy is involved in synaptic remodeling and plasticity in neurons^16,17^, disruption of autophagy may cause disturbed neuronal functions related to BPAN symptoms, such as psychomotor retardation and ID in early childhood. In this context, it is of note that a large-scale genomic study of individuals with undiagnosed and severe developmental disorders revealed de novo mutations in *WDR45*^18^. We thus explored the role of WDR45 in the developing and adult brain. We first showed that Wdr45 is distributed in excitatory synapses of cortical and hippocampal neurons by biochemical and morphological analyses, implicating a specific role of Wdr45 at post synapses. Then, since *WDR45* mutations responsible for BPAN are most likely to cause WDR45 instability^4^ and subsequent degradation, we carried out acute gene knockdown of Wdr45 by in utero electroporation in order to mimic the pathophysiological conditions of BPAN in mouse brain. Wdr45-deficiency was shown to cause defects in dendritic arborization as well as synaptogenesis in cortical excitatory neurons during brain development. We assume that the defects in synapse formation in cerebral cortex are responsible for a major BPAN symptom, ID. Regarding cortical axon development, interhemispheric extension was not affected at P2 and axons normally reached the contralateral hemisphere, whereas the pathfinding into the cortical layer structure and subsequent synapse network formation were hampered. These results also strongly suggest a pathophysiological role of *WDR45* gene abnormalities in disrupted synaptic function related to ID, although the underlying molecular mechanisms are yet to be elucidated.

*WDR45* gene abnormalities are thought to be responsible for other clinical features of BPAN including sudden-onset of progressive dementia and parkinsonism in adolescence. While the molecular pathophysiological mechanism remains largely unknown, several reports have shown that some BPAN symptoms in adolescence—adult phase, such as learning impairment and memory disturbance, are due to defective autophagy^6,10^. On the other hand, constitutive Wdr45-KO mouse demonstrated apoptosis-mediated neuronal loss in several brain regions, such as prefrontal cortex and basal ganglion in aged mouse^19^, whereas the CNS-specific Wdr45-KO mouse exhibited a less characteristic autophagy phenotype; no significant changes were observed in the number of neuronal cells in cerebellum and hippocampus^10^. Instead, these conditional KO mice displayed scattered remarkable axonal swelling in various brain regions including cerebral cortex and cerebellum, as well as reactive astrogliosis in various brain regions including hippocampus^10^. From these results, Wdr45-deficiency appears to be involved in varying degree of neuronal damage in the mouse brain, where increased ER (endoplasmic reticulum) stress and impaired ER quality control may be underlying molecular mechanism^19^. While morphological alteration was not reported in cerebral cortex of the 2 types of Wdr45-KO mice, it is notable that other members of the WIPI protein family (Wipi1–3) may compensate for the deficiency of Wdr45 in context-dependent manners. However, acute knockdown of Wdr45 caused defects in cortical neuron morphology in this study. We consider that acute knockdown by the combination of in utero electroporation and RNAi may circumvent the compensatory effects of general gene-knockout approaches. The limitation of the electroporation method may be the difficulty in analyses of aged mice because the RNAi vector is only transiently expressed in cortical neurons. In this context, in utero electroporation-mediated GFP-labeling of developing cortical neurons of the KO mice followed by fine morphological examination may provide in vivo data for better understanding the pathophysiology of BPAN.

Pathophysiological significance of autophagy in the abnormal morphological phenotypes observed in this study remains to be clarified. While disruption of ER function may be involved, further intensive investigation is needed to clarify the molecular basis of the functional defects of WDR45 as well as the relationship between hampered autophagy and BPAN etiology. In addition, given that some autophagy proteins participate in autophagy-independent processes^20^, results in this study might suggest dysregulation of yet unidentified Wdr45 function(s) involved in BPAN, although further studies are again required to address this possibility.

In conclusion, the present study suggests a role for Wdr45 in dendritic arborization, synaptogenesis and axon network formation of cortical excitatory neurons. We propose that WDR45-deficiency may be associated with BPAN neurodevelopmental symptoms by affecting the function of yet unidentified protein(s) involved in cytoarchitecture formation/maintenance such as cytoskeletal proteins and other linked cell signaling molecules. Further investigation will be essential to clarify the relationship between the pathophysiological mechanism of *WDR45* gene abnormalities and the clinical features of BPAN.

## Materials and methods

### Ethics approval and consent to participate

We followed the fundamental guidelines for proper conduct of animal experiments in academic research institutions under the jurisdiction of the Ministry of Education, Culture, Sports, Science and Technology, Japan. All the protocols for animal handling and treatment were reviewed and approved by the animal care and use committee of Institute for Developmental Research, Aichi Developmental Disability Center (approval number M-10). All experiments were conducted in compliance with ARRIVE guidelines. All methods, data presentation and statistical analyses were carried out in accordance with the ‘TOP’ guidelines. This study has not been pre-registered.

### Plasmids

Mouse (m)*Wdr45* was amplified by PCR and subcloned into pCAG-Myc vector (Addgene Inc., Cambridge, MA). For RNAi experiments, following target sequences were inserted into pSuper-puro vector (OligoEngine, Seattle, WA): mWdr45#1, CAAGAAAGCTGTTTGAGTT (395–413); mWdr45#2, CCCTTATTCGTCTCTTTGA (644–662). Numbers indicate the positions from Wdr45 translational start sites. We named these vectors as pSuper-Wdr45#1 and #2. For the control RNAi experiments, pSuper-H1.shLuc designed against luciferase (CGTACGCGGAATACTTCGA) was used. To generate RNAi-resistant Wdr45, Wdr45R, silent mutations were introduced, as underlined, in the target sequence (CAAGGAAATTATTCGAGTT in mWdr45#1). The Wdr45 mutant related to BPAN pathogenicity^7^, c.700C > T/p.(Arg234*) (Wdr45-Rstop), was prepared using KOD-Plus Mutagenesis kit (Toyobo Inc., Osaka, Japan). Likewise, another mutant with a single amino acid substitution, c.439G > T/p.(Gly167Val) (Wdr45GV), was prepared. For antibody absorption experiments, full length *Wdr45* was subcloned into pMal-p2, expressed in *E. coli* and affinity-purified as a recombinant protein according to the manufacturer’s instruction (New England BioLabs Inc., Ipswich, MA). All constructs were verified by DNA sequencing.

### Primary antibodies

Polyclonal rabbit anti-Wdr45 antibody was kindly provided by Dr. H. Saitsu (Hamamatsu Medical University, Japan)^4^. Polyclonal rabbit anti-GFP and anti-Myc were prepared and characterized previously^21^. Polyclonal rabbit anti-Sept11 was prepared as described^22^. Mouse monoclonal anti-GFAP (Sigma-Aldrich, St. Louis. MO, USA), anti-synaptophysin (Progen Biotechnik, Heidelberg, Germany), anti-PSD95 (Thermo Scientific, Waltham, MA) and anti-Myc (Cell signaling, Nagoya, Japan) antibodies were also used.

### Preparation of extracts from mouse tissues and western blotting

Sample preparation was performed essentially as described^23–25^. Briefly, tissues from ICR adult mice (Japan SLC, Shizuoka, Japan) were homogenized with the lysis buffer (50 mM Tris–HCl, pH 7.5, 0.1% NaF, 5 mM EDTA, 1 mM Na_3_VO_4_ and the protease inhibitor mix). Each suspension was sonicated on ice and solubilized with the lysis buffer containing 2% SDS. Protein concentration was estimated with BCA protein assay reagent kit (Pierce, Rockford, IL) with bovine serum albumin as a standard. SDS-PAGE (10% gel) and western blotting were performed as described^23–25^.

### Preparation of postsynaptic density (PSD) fractions

Sample preparation was performed essentially as described^24^. Briefly, 5 adult mouse brains were homogenized in 23 ml of 5 mM HEPES (pH 7.4) containing 1 mM MgCl_2_, 0.5 mM CaCl_2_, phosphatase inhibitors and the protease inhibitor mix with a Teflon homogenizer (H fraction). The resulting extract was centrifuged at low speed (1400×*g* for 10 min) to obtain S1 as a supernatant fraction. S2 fraction was then obtained by centrifugation of S1 at 13,800×*g* for 10 min. The resulting pellet (P2) was resuspended in 0.25 ml of 6 mM Tris (pH 8.0) containing 0.32 M sucrose, loaded onto a discontinuous sucrose gradient, and centrifuged at 82,500×*g* for 2 h. The synaptosome fraction was collected, extracted with 0.5% Triton X-100 and centrifuged at 32,800×*g* for 20 min. The resulting pellet (PSD-I; crude PSD fraction) was again extracted with 0.25 ml of 0.5% Triton X-100 and centrifuged again at 201,800×*g* for 1 h to obtain highly purified PSD fraction as pellet (PSD-II) which was dissolved in 0.25 ml of SDS-PAGE sample buffer.

### Immunohistochemical analyses

Mouse brains of either sex were fixed with 4% paraformaldehyde at various time points and cut into coronal section (6-µm thickness) at the level of dorsal hippocampus. After deparaffinization, sections were processed for immunohistochemistry as reported previously^22,23^. Images were captured using BZ-9000 microscope (Keyence Inc., Osaka, Japan).

### Cell culture, transfection and immunofluorescence

COS7 cell transfection experiments were performed with polyethyleneimine “MAX” reagent **(**Polysciences Inc., Warrington, PA**)**. Immunofluorescence analyses were carried out as described^23–25^. Alexa Fluor 488-, 594- or 647-labeled IgG (Life Technologies) was used as a secondary antibody. Fluorescent images were captured with a Zeiss880 confocal microscopy. Primary cultured cortical neurons and astrocytes were isolated from E14.5 mouse embryos as described^23,26,27^. Briefly, dissected cortices were mechanically and enzymatically disrupted in the presence of trypsin and DNase I for 15 min at 37 ℃. Neurons were then cultured in Neurobasal medium with B27 supplement (Thermo scientific, Waltham, MA), 1 mM l-glutamine, 100 U/ml penicillin and 100 µg/ml streptomycin. Astrocytes were then cultured in the DMEM medium.

### In utero electroporation, and analyses of neuronal migration and axon elongation

In utero electroporation was carried out at E14.5 and analyses were conducted at P2 essentially as described^23^. E14.5 ICR mice were housed individually given ad libitum access to food and water. Both female and male pups were used. Pups were maintained in a 12:12 light–dark cycle together with their dams before being sacrificed at indicated time points, which were primary endpoints of this study. At least 5 electroporated brains from different dams were used for each experiment. After anesthetization of pups on ice for 5 to 10 m affording physical relief, brains were fixed overnight in 4% paraformaldehyde phosphate buffer solution. Sections (100 μm- and 150 μm-thickness for P2 and above P10 samples, respectively) were prepared by vibratome. Distribution of GFP-positive neurons was then analyzed as described^28,29^. Notably, neuronal progenitor cells present in the VZ at E14.5 are to become layer II/III pyramidal neurons in the adult stage. Thus, progenitor cells electroporated at E14.5 are to make it to the layer II/III under the normal conditions. Also, given that transfection efficiency into each cell depends on the size of the cell surface area which is physically exposed to the ventricular lumen (cerebrospinal fluid) where plasmids are injected, neurons incorporating low amount of a vector are supposed to undergo partial effects.

For estimation of axon growth, GFP signal intensity of the callosal axons was measured in a 200 × 200 µm rectangle on both the ipsilateral and contralateral sides at the positions indicated in Fig. 5A. The ratio of the axonal RFP signals in the contralateral side to the corresponding ipsilateral side was calculated.

### Quantitative analysis of dendritic arbor formation

To measure the length and branching point number of dendrites of post-migratory (mature) neurons, images of GFP-positive neurons in the layer II–III of cerebral cortex at P10, P30, P60 or P90 were acquired by a Zeiss880 confocal microscopy. Image J software was used for the quantitative analyses of dendritic length and Sholl test.

### Quantitative analysis of spine morphology

Electroporated neurons were visualized by immunostaining of co-transfected GFP with anti-GFP. Electroporated cells were chosen randomly and images were obtained using a Zeiss880 confocal microscope. We took z-series stacks (0.2 μm-thickness) and used Zen software to generate image projections for quantitative analyses. Morphometric measurements were automatically performed using ImagePro PLUS software. To analyze spine density and morphology, more than 200 spines from 20 or more neurons were measured for each condition. Morphological assessments were conducted blindly.

### Statistical analysis

Data were analyzed using GraphPad Prism 8 software (GraphPad Software Inc., La Jolla, CA). Results were expressed as means ± SD. When data were obtained from only 2 groups, Student’s *t*-test was used for comparison. Values are expressed as means ± SEM. For other experiments, the rate of cell scores was initially analyzed using the one-way analysis of variance (ANOVA) with Tukey’s or Dunnett’s post hoc test for multiple comparisons. The level of statistical significance was considered to be *p* < 0.05.

## Supplementary Information

Supplementary Figures.

## Data Availability

All data generated or analyzed during this study are included in this published article and its supplementary information files.
