# Supplementary material for: Physiological significance of WDR45, a responsible gene for β-propeller protein associated neurodegeneration (BPAN), in brain development
Source: Sci Rep. 2021 Nov 19;11:22568. doi: 10.1038/s41598-021-02123-3 (PMC8604945; doi:10.1038/s41598-021-02123-3)
Supplement: Supplementary file 1 — Supplementary Figures. [file 41598_2021_2123_MOESM1_ESM.pdf]

Supplemental Information

Physiological significance of *WDR45*, a responsible gene for β-propeller protein associated neurodegeneration (BPAN), in brain development

Mariko Noda[1], Hidenori Ito[1] and Koh-ichi Nagata[1,2*]

[1]Department of Molecular Neurobiology, Institute for Developmental Research, Aichi Developmental Disability Center, Kasugai
[2]Department of Neurochemistry, Nagoya University Graduate School of Medicine, Nagoya, Japan
[*]Corresponding author

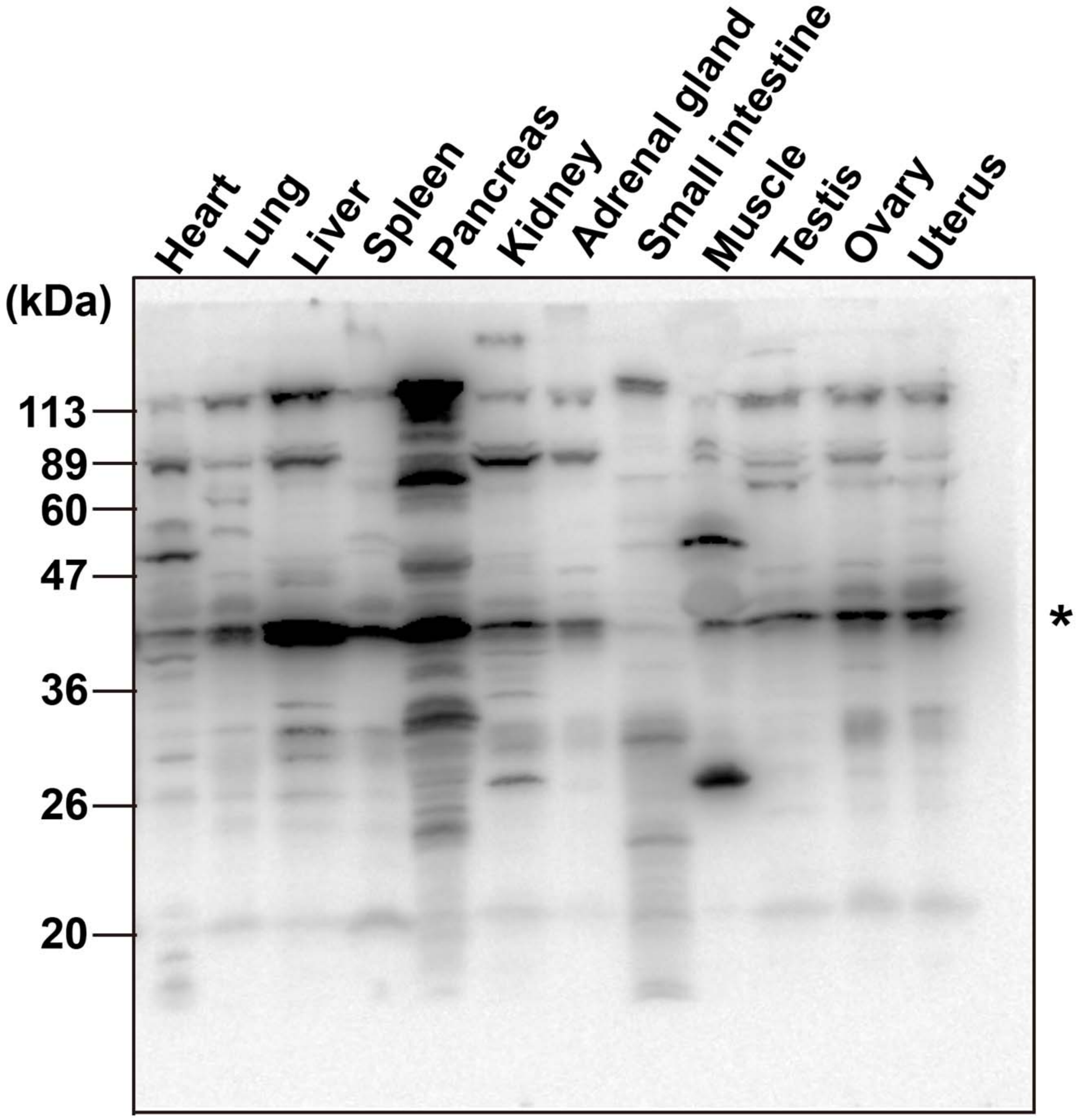

Supplementary Figure 1. Wdr45 protein expression profile in mouse body

Whole lysates (30 µg protein per lane) of various adult mouse tissues were subjected to western blotting (15 % gel) with anti-Wdr45. Asterisk denotes a band of expected size of Wdr45 protein. Molecular weight markers are shown at the *left*.

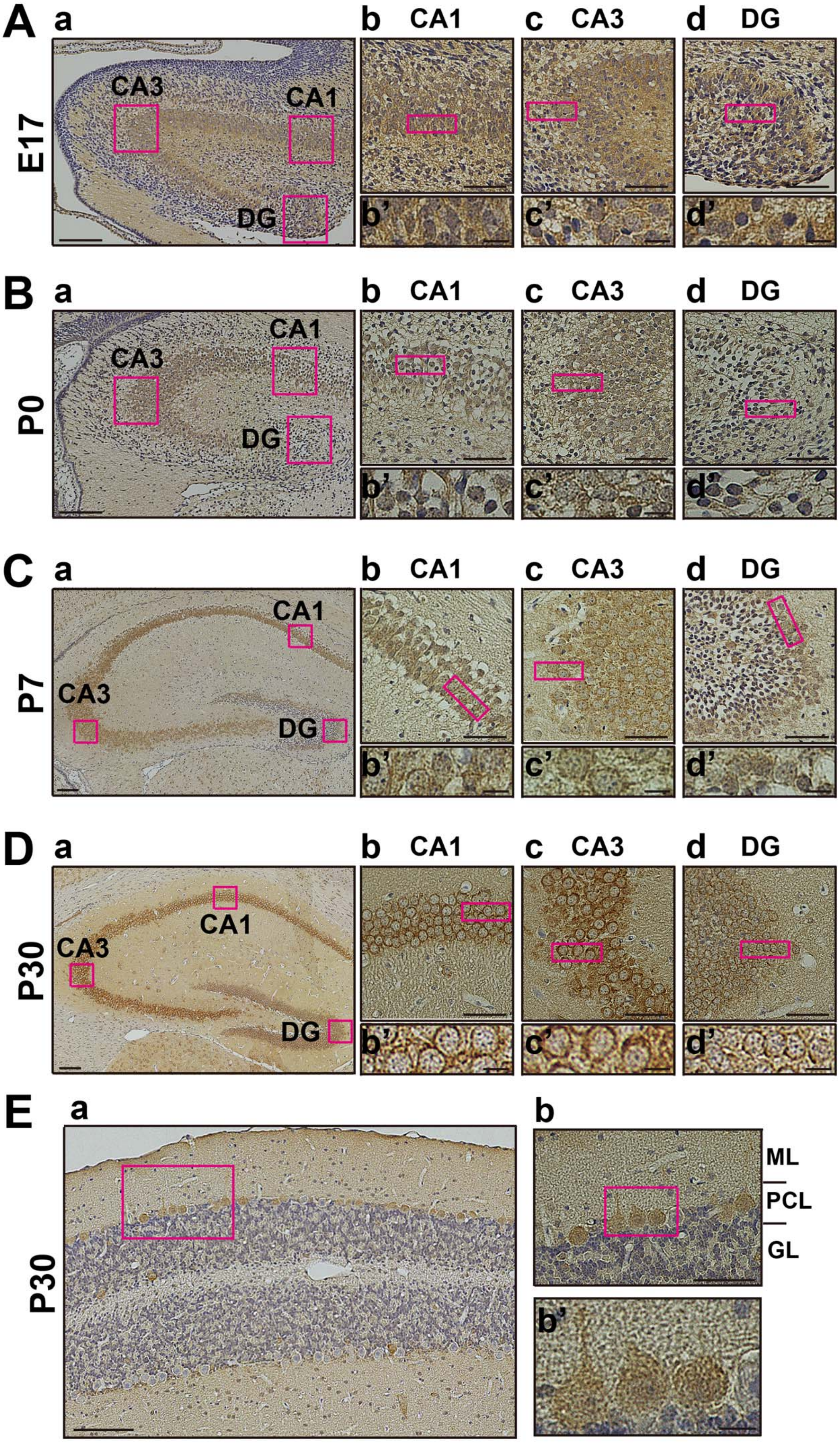

A
a
b CA1
c CA3
d DG
E17
CA3
CA1
DG
b'
c'
d'
B
P0
C
P7
D
P30
E
ML
PCL
GL

Supplementary Figure 2. Immunohistochemical analyses of Wdr45 in hippocampus and cerebellum

Hippocampal sections at E17 **(A)**, P0 **(B)**, P7 **(C)** and P30 **(D)** were stained with anti-Wdr45. Boxed areas in (a) were magnified in (b) – (d). Boxed areas in (b) – (d) were magnified in (b') – (d'), respectively. CA, *cornu ammonis*; DG, *dentate gyrus*. **(E)** A cerebellar section at P30 was stained with anti-Wdr45. Boxed areas in (a) and (b) were magnified in (b) and (b'), respectively. Layers were indicated in (b). ML, *molecular layer*; PCL, *Purkinje cell layer*; GL, *granular layer*. Counter staining was done with Mayer's hematoxylin. Scale bars; 100 μm (A-E,a), 50 μm (A-D,b-d and E,b) and 10 μm (A-D,b'-d' and E,b').

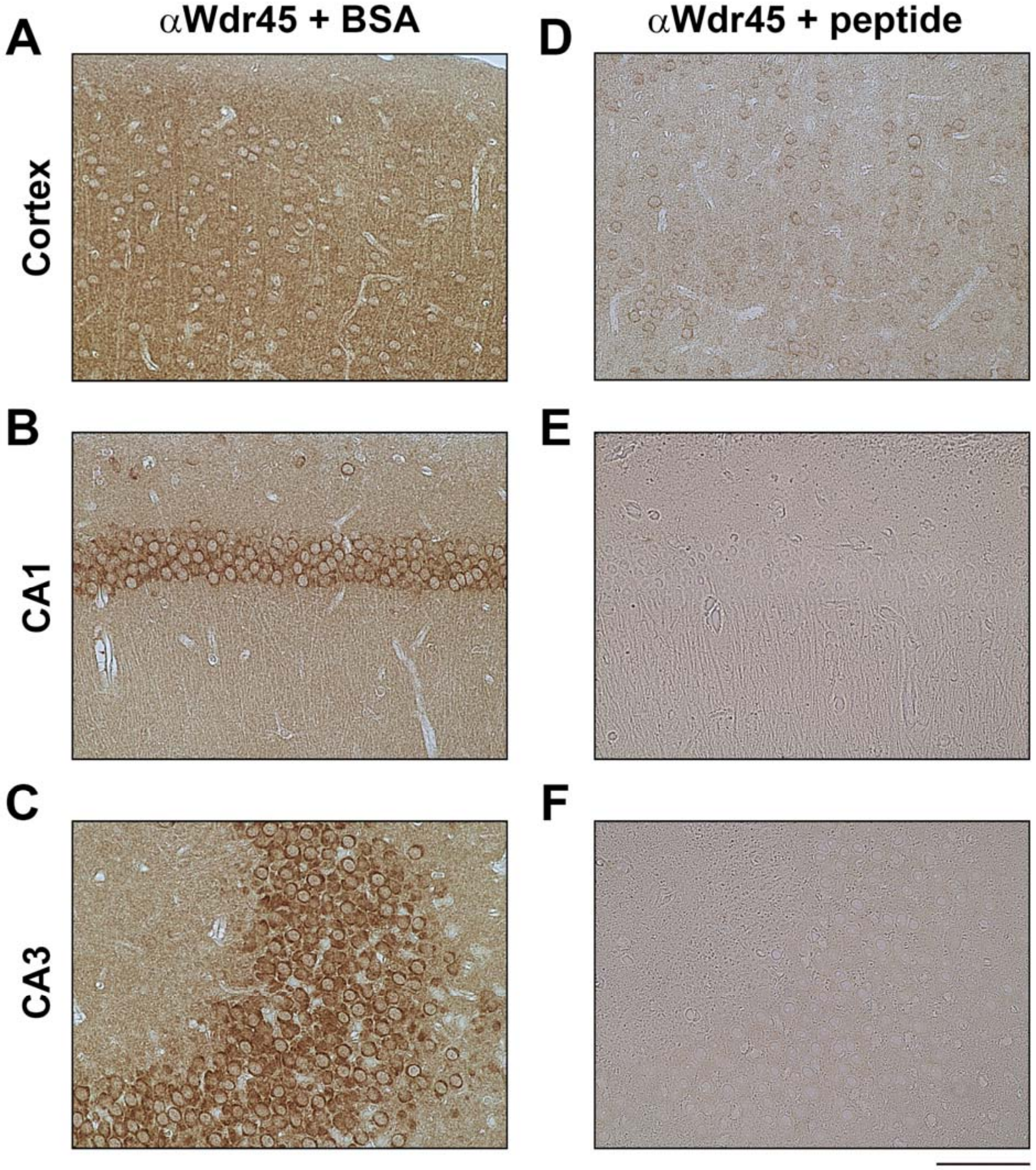

Supplementary Figure 3. Specificity of anti-Wdr45 was determined by absorption with recombinant Wdr45.

Anti-Wdr45 was pre-absorbed without (A-C) or with excess amount of recombinant Wdr45 (D-F). The recombinant protein was prepared as described in “Materials and methods” section. Resultant antibody was used for immunostaining with sections of cerebral cortex (A, C), hippocampal CA1 (B, D) or CA3 (C, F) from P30 mouse brain. Scale bar, 100 μm.

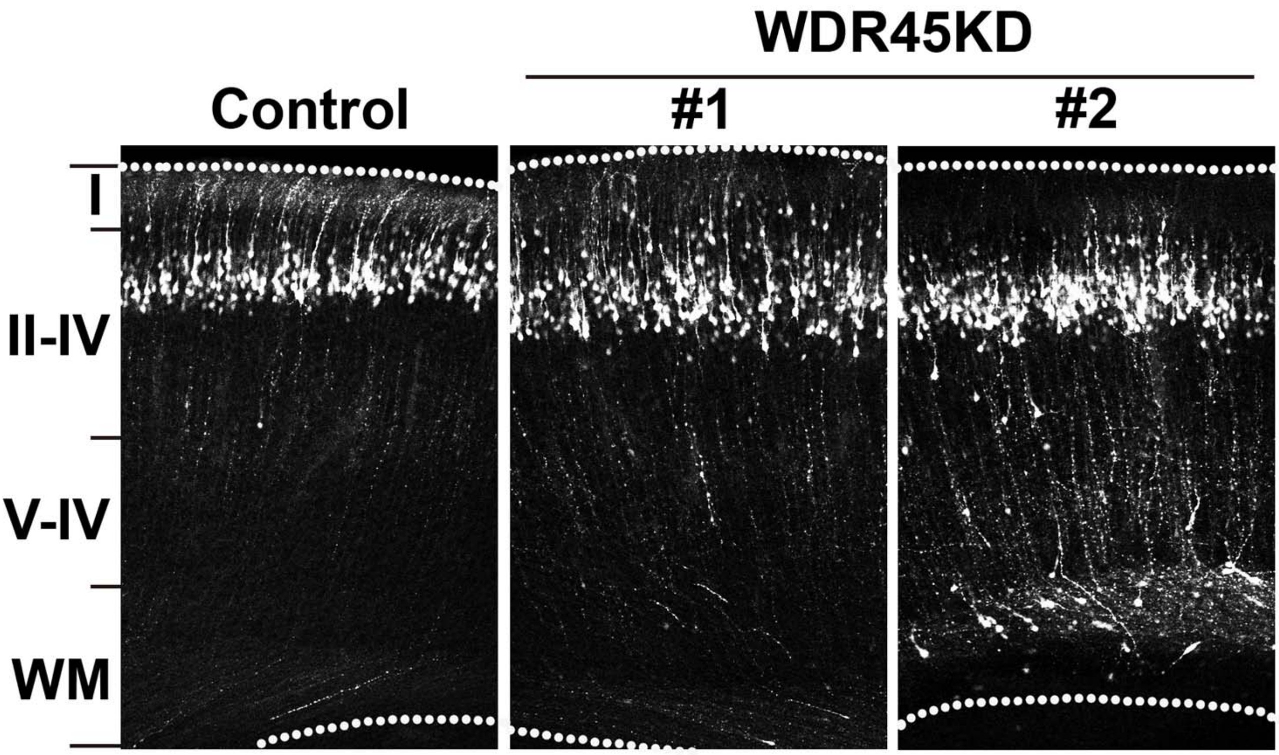

Supplementary Figure 4. Effects of Wdr45-knockdown on cortical neuron migration during corticogenesis.
pCAG-TurboRFP was co-electroporated with pSuper-H1.shLuc (control) or pSuper-Wdr45#1 or #2 into the VZ progenitors at E14.5. Coronal sections were prepared at P2 and stained for RFP. Dotted lines represent the pial (*upper*) and the ventricular (*lower*) surfaces.

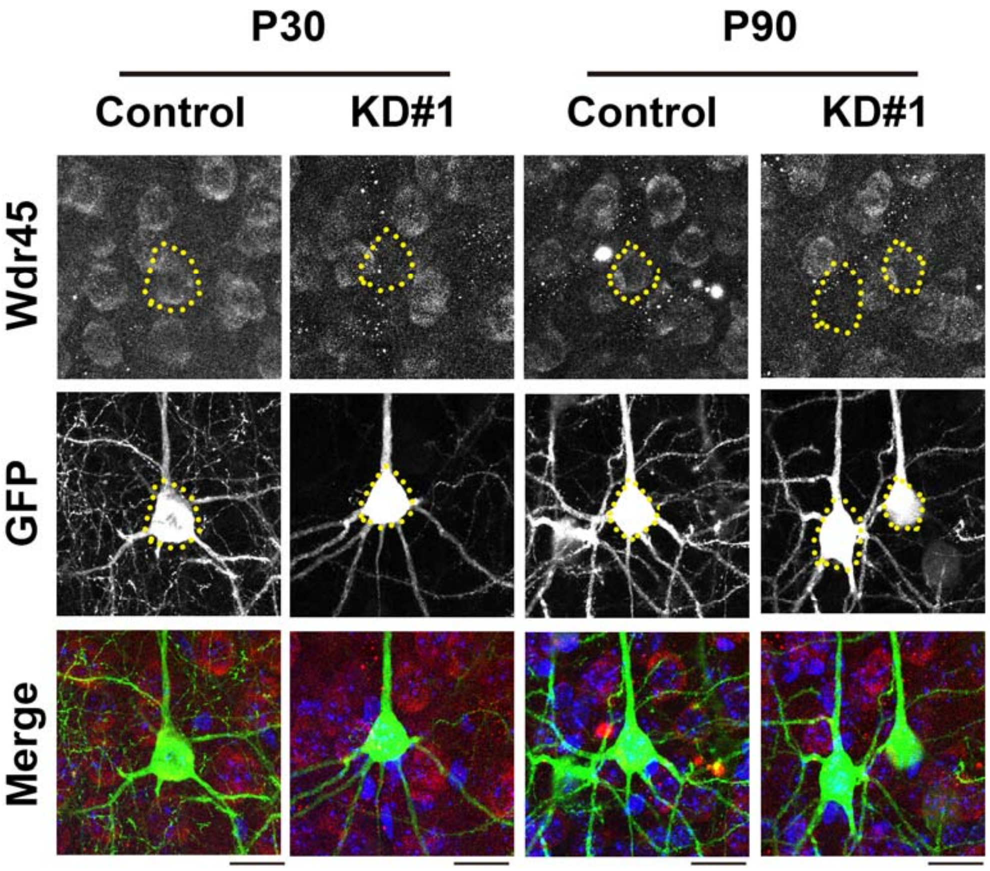

Supplementary Figure 5 Knockdown of endogenous Wdr45 in adult mouse brain

pCAG-loxP-GFP was co-electroporated with pCAG-M-Cre together with pSuper-H1.shLuc (Control) or pSuper-Wdr45#1 (KD#1) at E14.5. After fixation at P30 or P90, coronal sections were stained for GFP and Wdr45. GFP-positive cell soma regions were circled with yellow dotted lines. Scale bars, 5 μm.

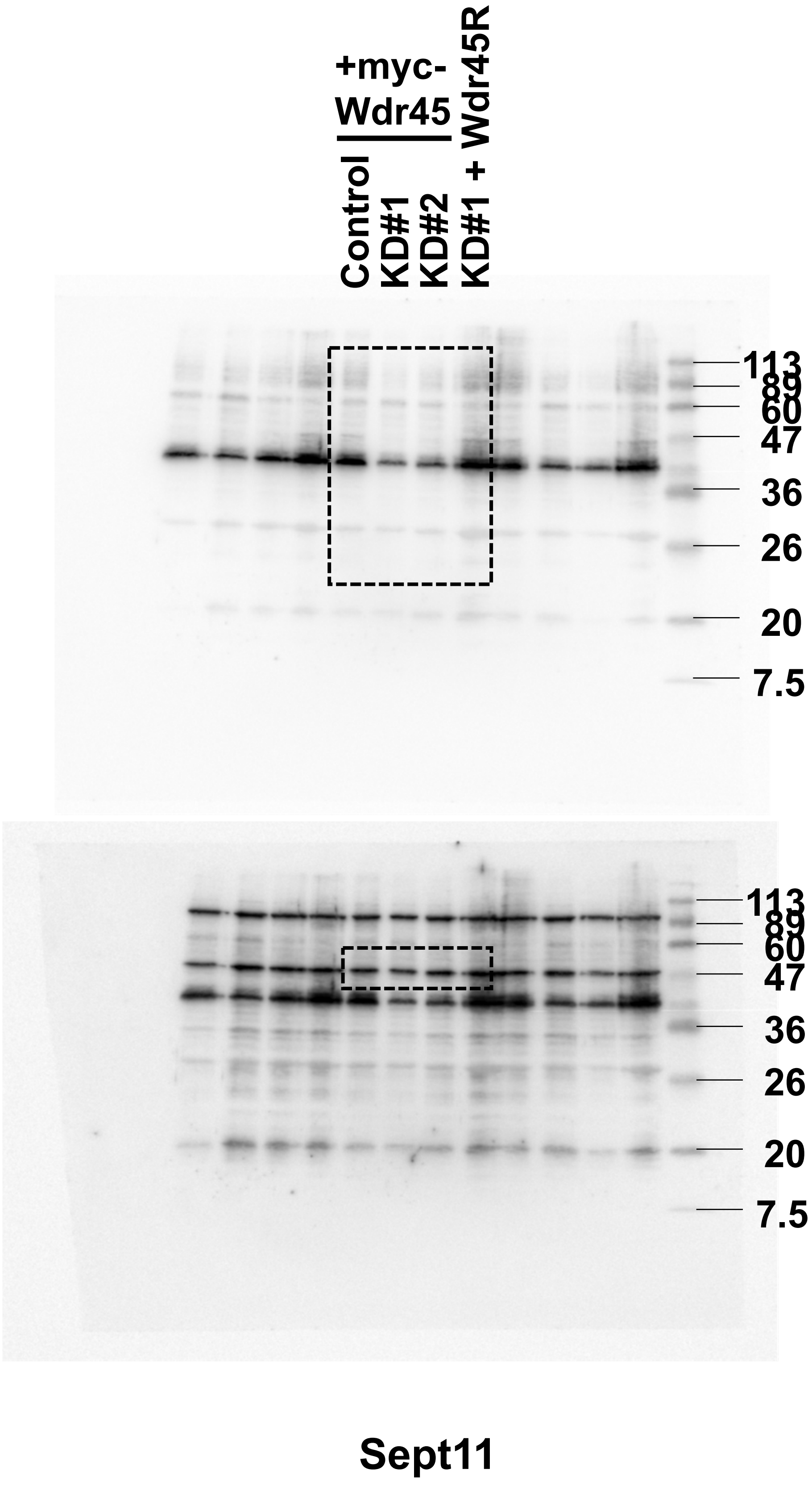

Supplementary Figure 6. Uncropped blots of Figure 1A

The protein bands shown in Figure 1A are indicated by *boxed lines*.

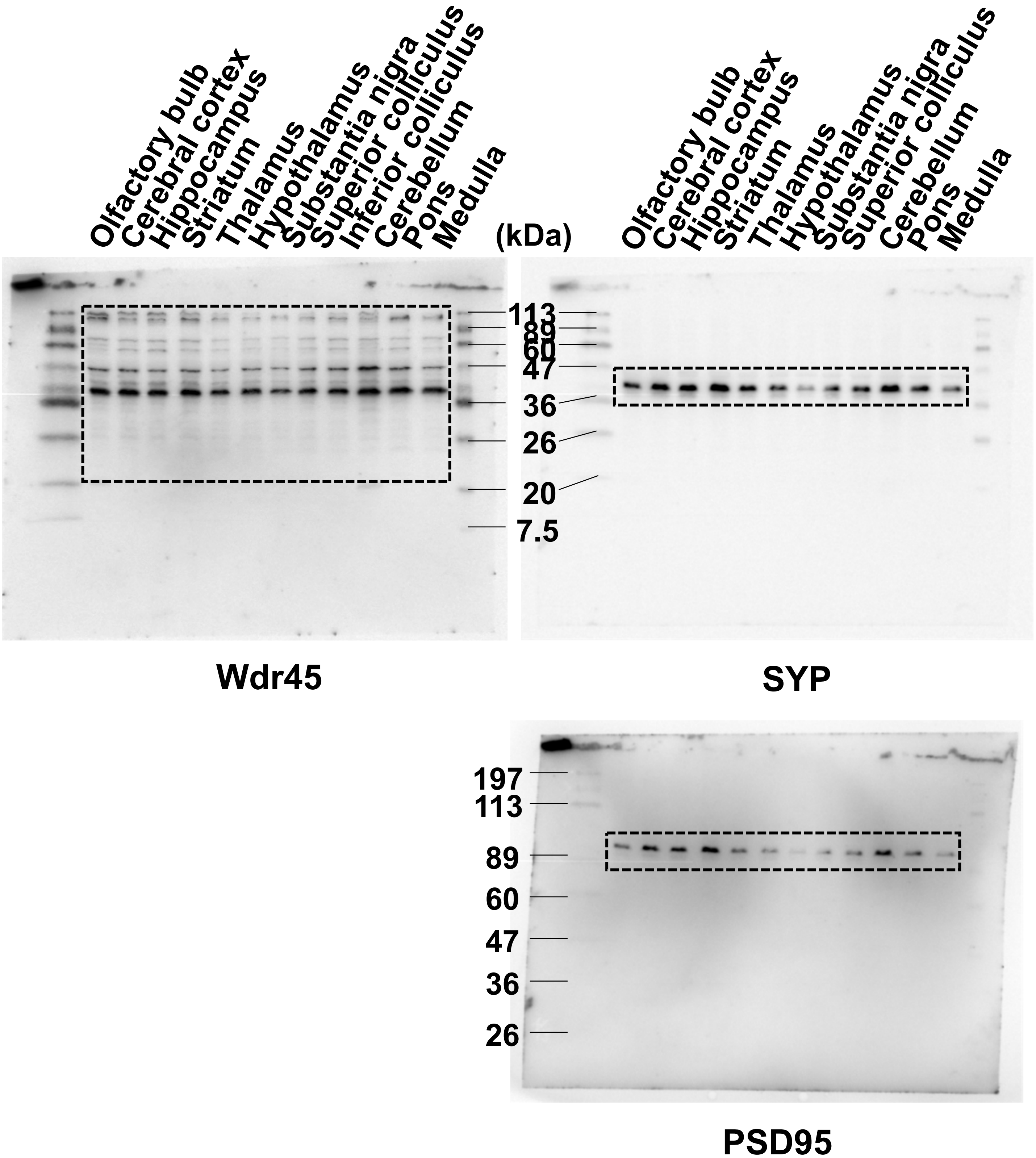

Supplementary Figure 7. Uncropped blots of Figure 1B

The protein bands shown in Figure 1B are indicated by *boxed lines*.

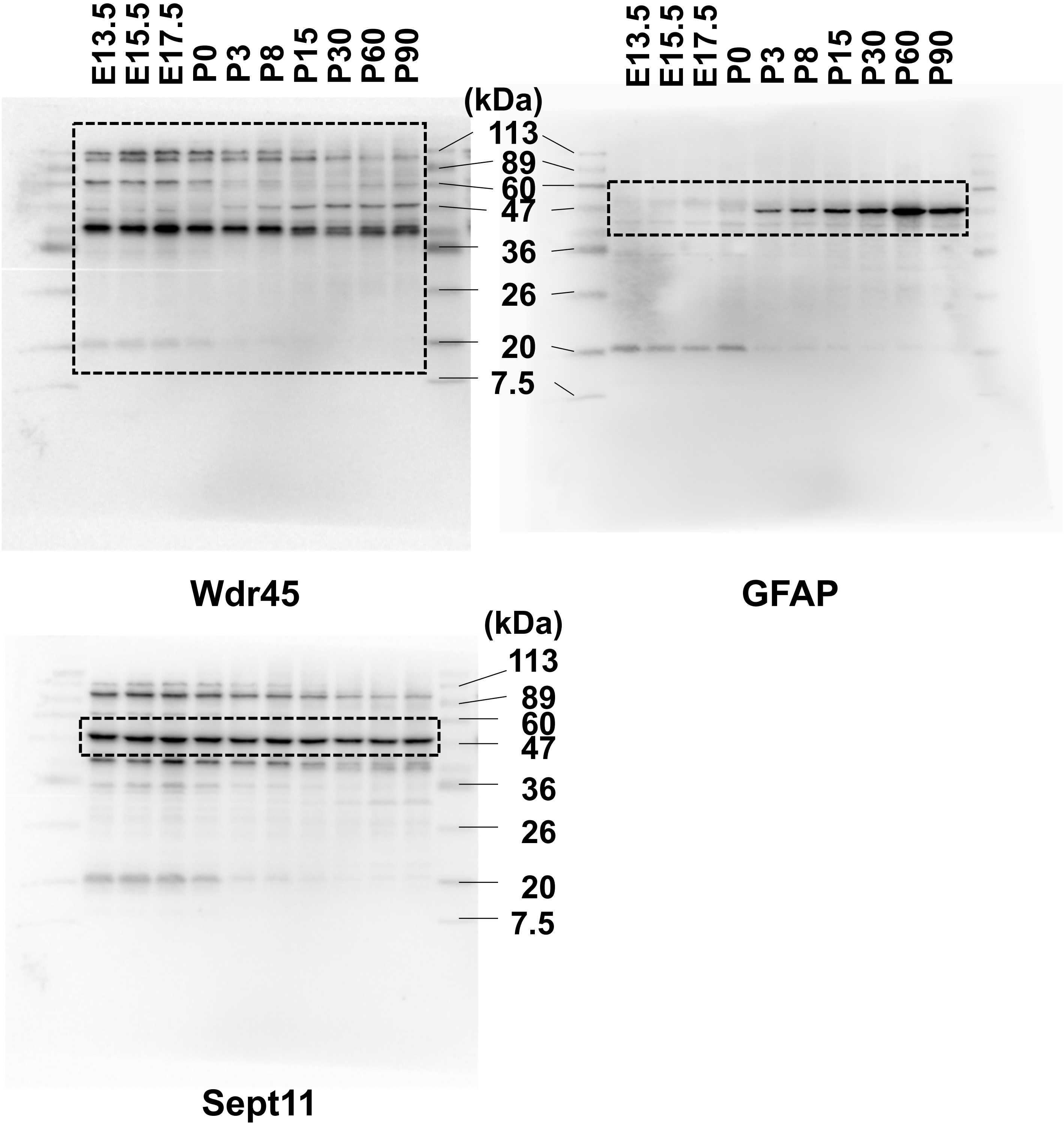

Supplementary Figure 8. Uncropped blots of Figure 1C

The protein bands shown in Figure 1C are indicated by *boxed lines*.

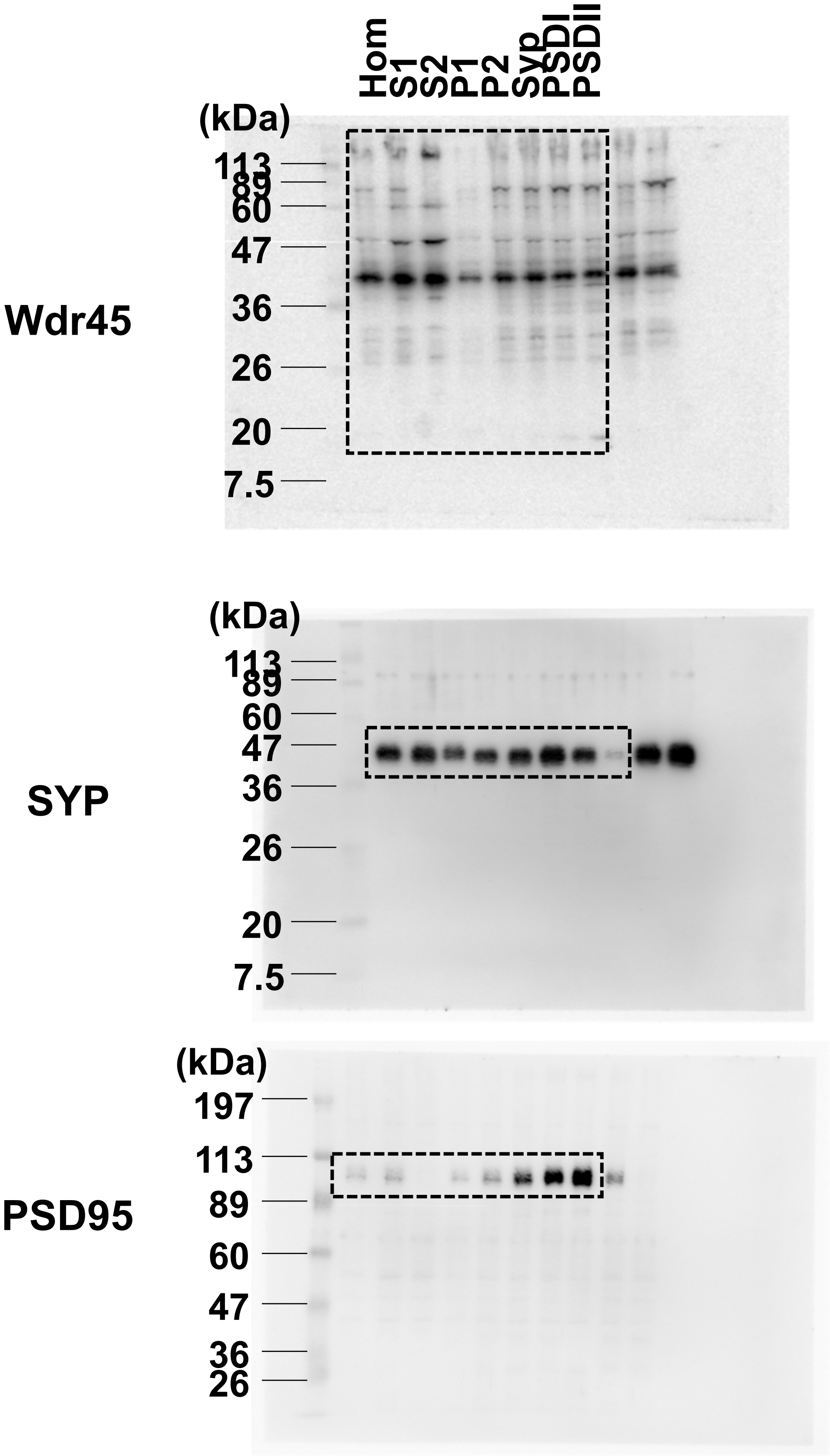

Supplementary Figure 9. Uncropped blots of Figure 1D

The protein bands shown in Figure 1D are indicated by *boxed lines*.

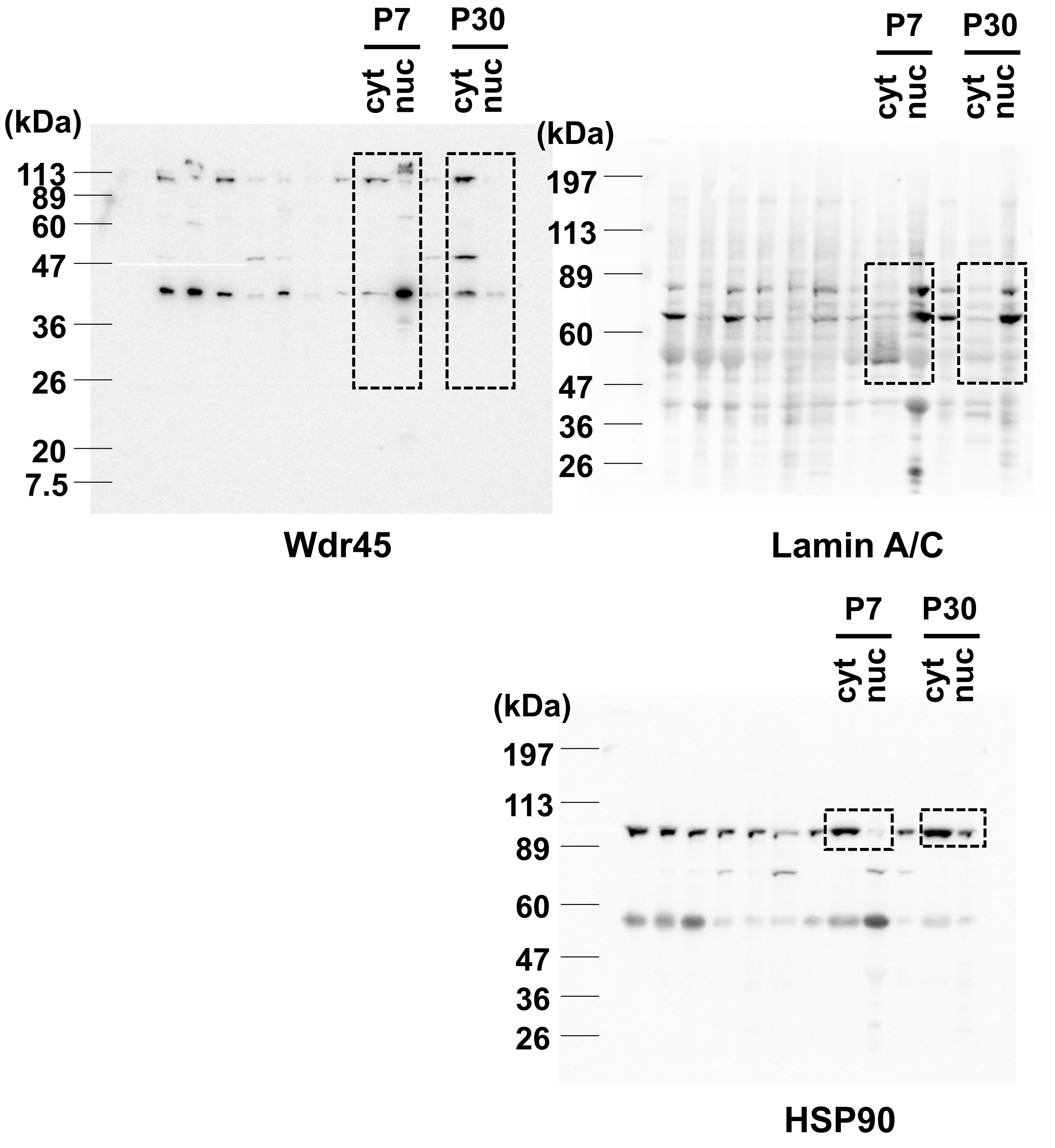

Supplementary Figure 10. Uncropped blots of Figure 2E

The protein bands shown in Figure 2E are indicated by *boxed lines*.

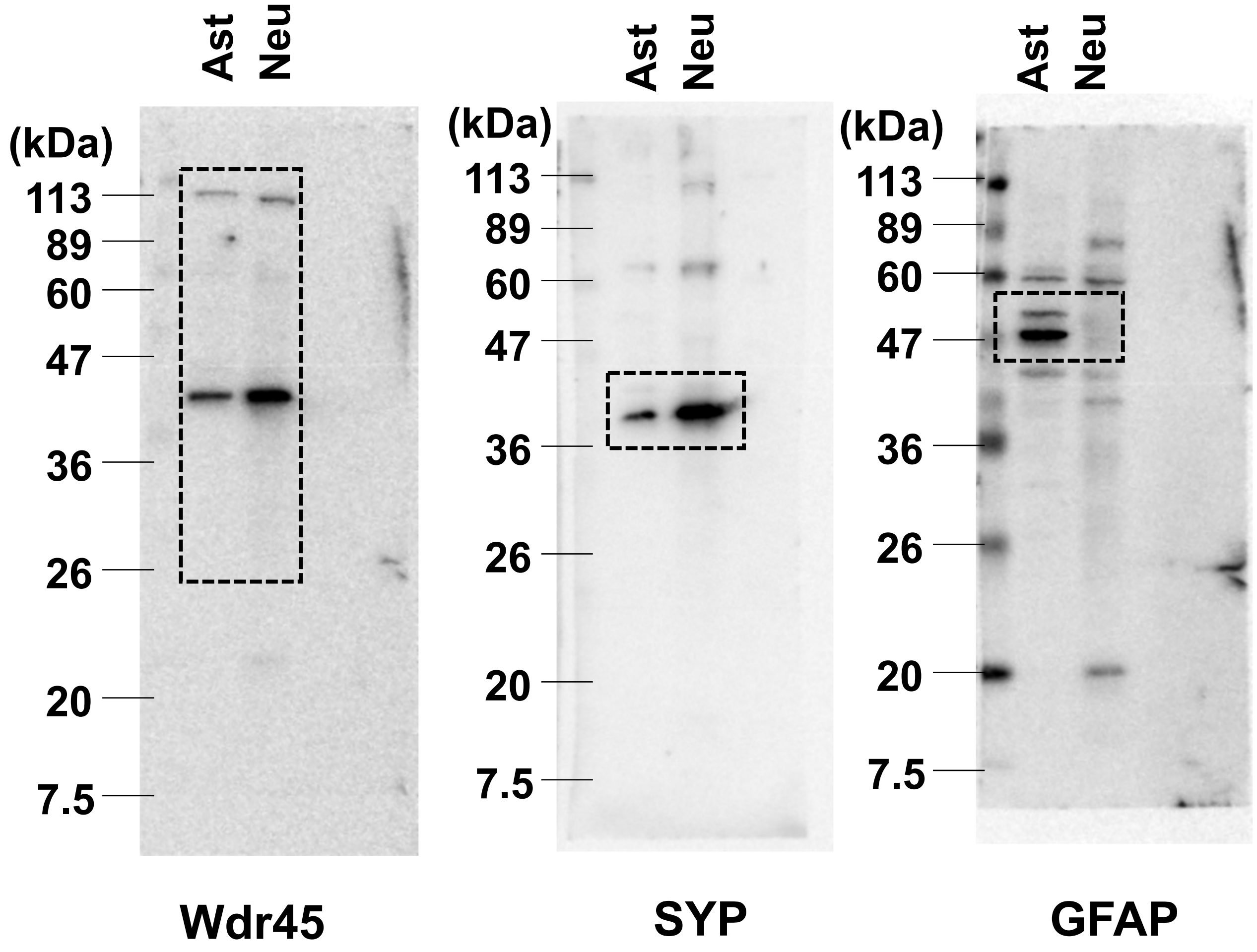

Supplementary Figure 11. Uncropped blots of Figure 2F
The protein bands shown in Figure 2F are indicated by *boxed lines*.

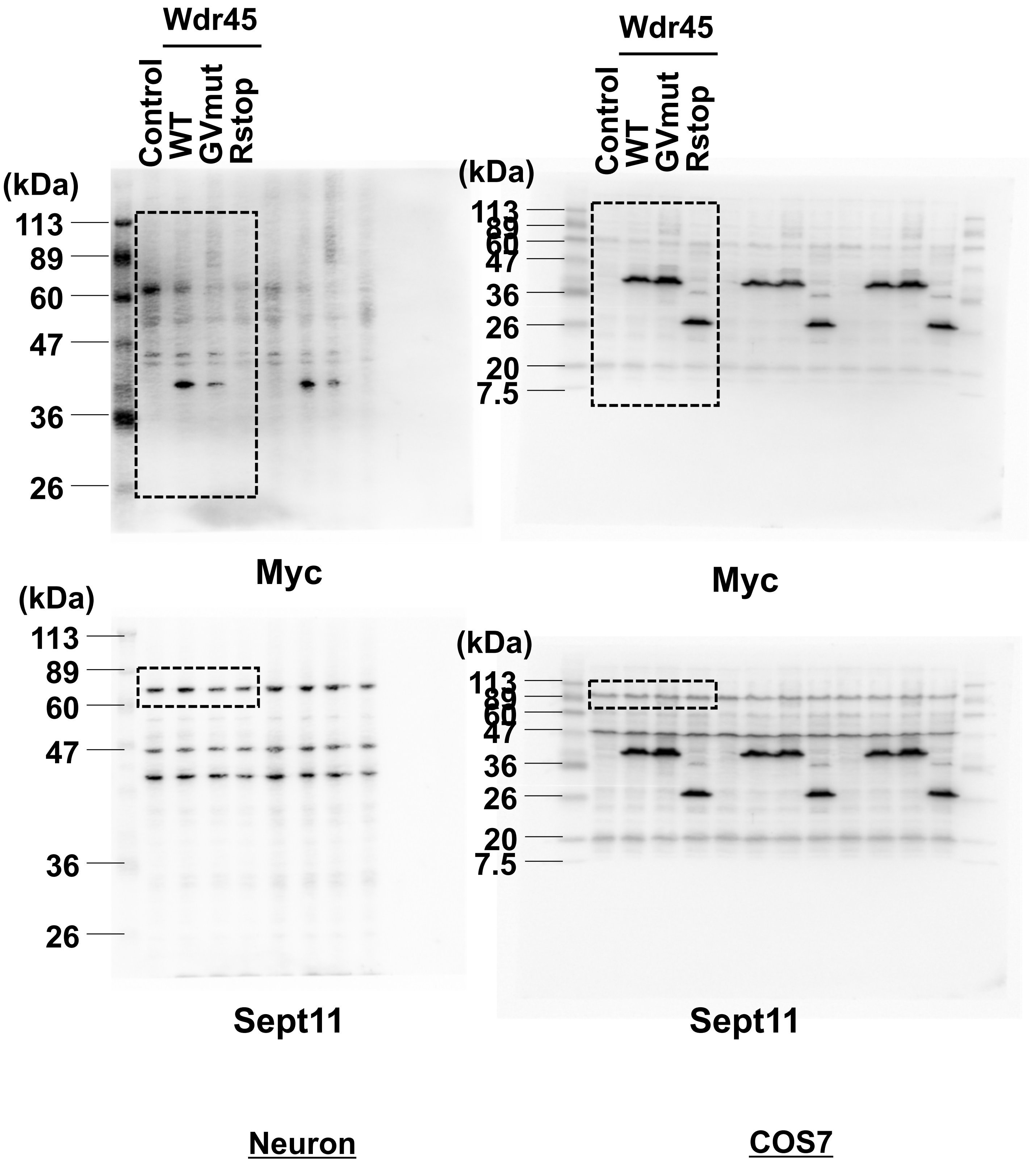

Supplementary Figure 12. Uncropped blots of Figure 4A
The protein bands shown in Figure 4A are indicated by *boxed lines*.

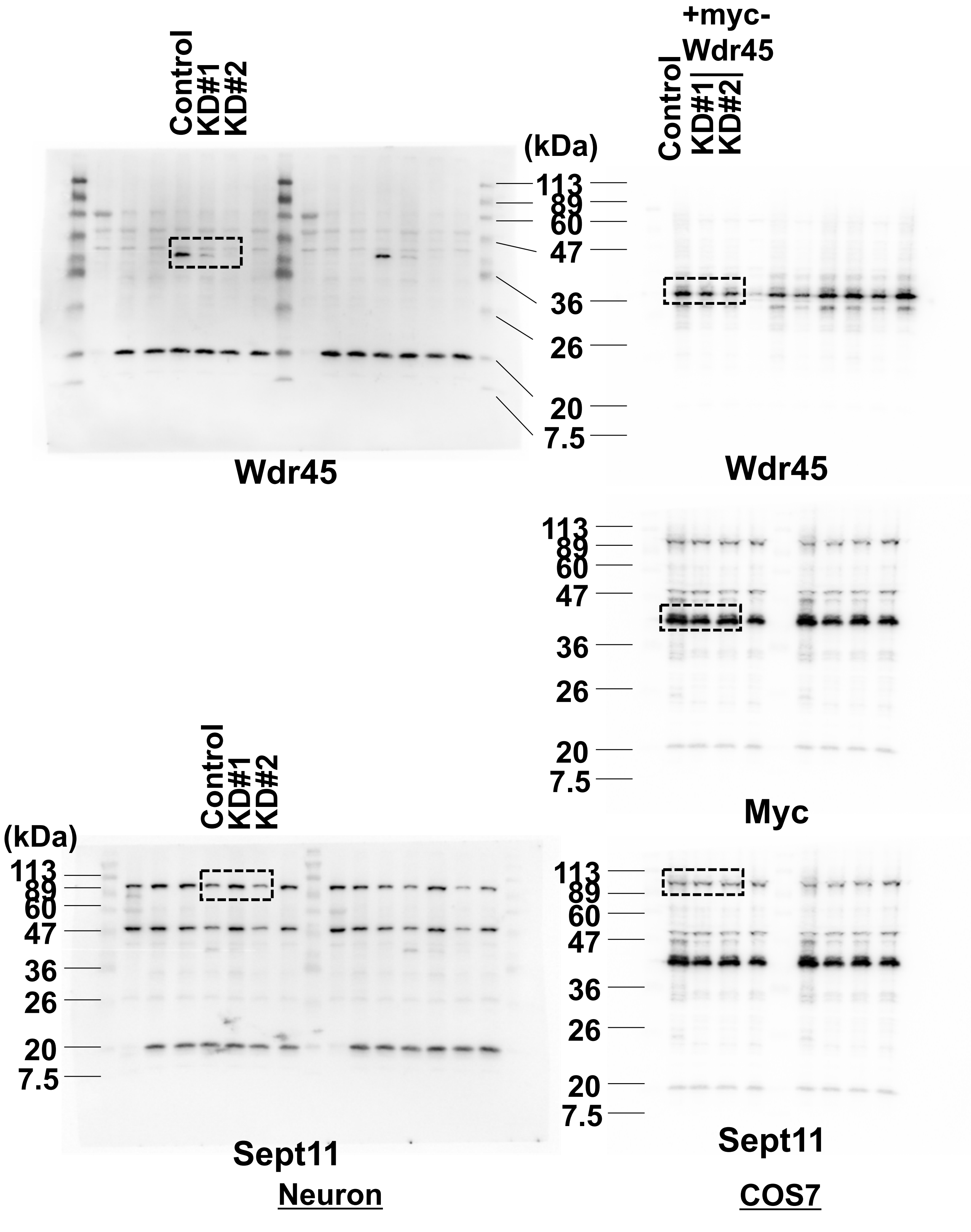

Supplementary Figure 13. Uncropped blots of Figure 4B

The protein bands shown in Figure 4B are indicated by *boxed lines*.

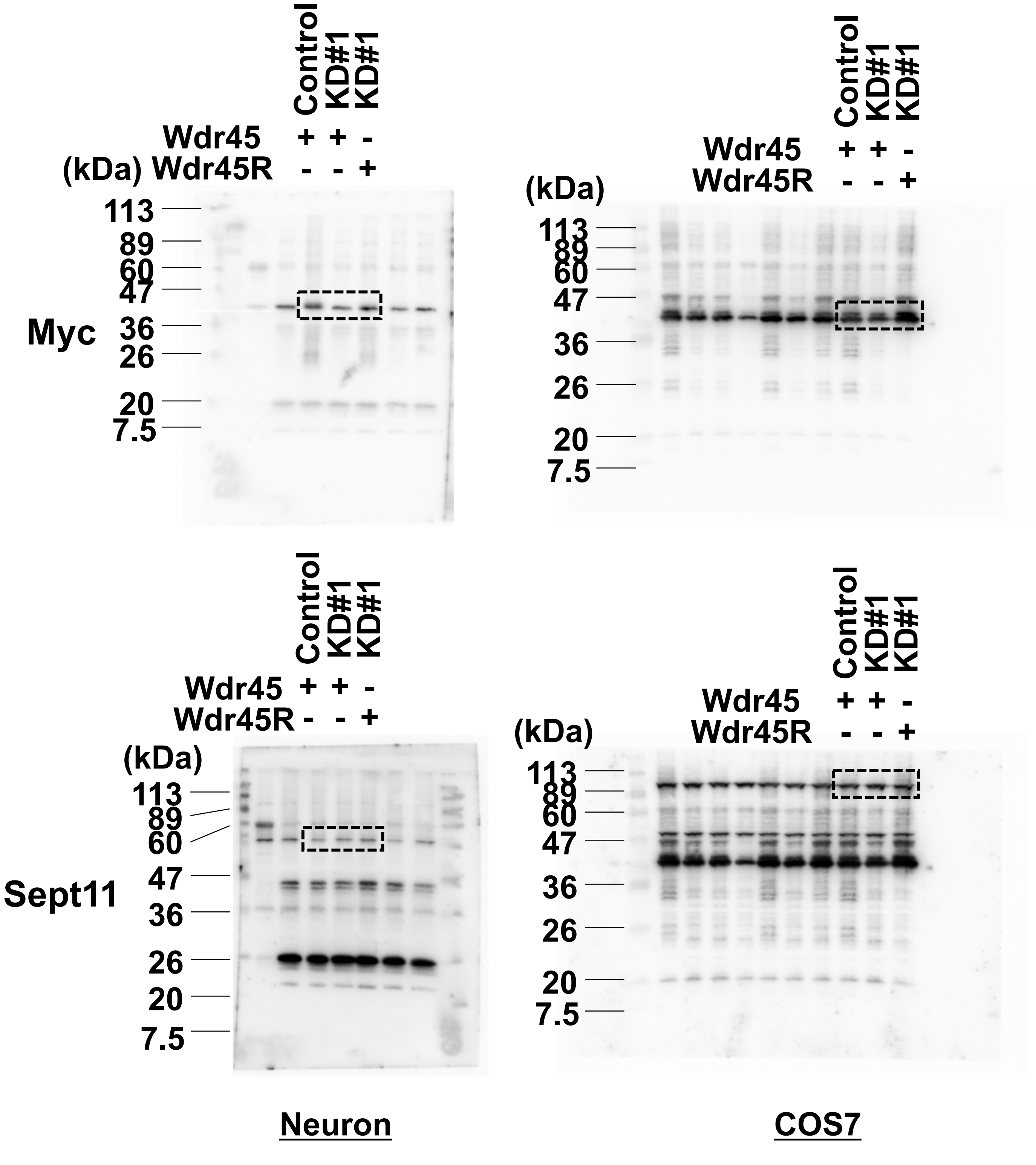

Supplementary Figure 14. Uncropped blots of Figure 4D
The protein bands shown in Figure 4D are indicated by *boxed lines*.
